# Use of a small DNA virus model to investigate mechanisms of CpG dinucleotide-induced attenuation of virus replication

**DOI:** 10.1099/jgv.0.001477

**Published:** 2020-08-12

**Authors:** Lisa Loew, Niluka Goonawardane, Jeremy Ratcliff, Dung Nguyen, Peter Simmonds

**Affiliations:** ^1^​ Nuffield Department of Medicine, Peter Medawar Building for Pathogen Research, University of Oxford, Oxford OX1 3SY, UK; ^†^​ Present address: Clinical Biomanufacturing Facility, University of Oxford, Old Road, Headington, Oxford OX3 7BN, UK

**Keywords:** parvovirus, CpG dinucleotide, TpA dinucleotide, zinc antiviral protein, codon optimization, minute virus of mice

## Abstract

Suppression of the CpG dinucleotide is widespread in RNA viruses infecting vertebrates and plants, and in the genomes of retroviruses and small mammalian DNA viruses. The functional basis for CpG suppression in the latter was investigated through the construction of mutants of the parvovirus, minute virus of mice (MVM) with increased CpG or TpA dinucleotides in the VP gene. CpG-high mutants displayed extraordinary attenuation in A9 cells compared to wild-type MVM (>six logs), while TpA elevation showed no replication effect. Attenuation was independent of Toll-like receptor 9 and STING-mediated DNA recognition pathways and unrelated to effects on translation efficiency. While translation from codon-optimized VP RNA was enhanced in a cell-free assay, MVM containing this sequence was highly attenuated. Further mutational analysis indicated that this arose through its increased numbers of CpG dinucleotides (7→70) and separately from its increased G+C content (42.3→57.4 %), which independently attenuated replication. CpG-high viruses showed impaired NS mRNA expression by qPCR and reduced NS and particularly VP protein expression detected by immunofluorescence and replication in A549 cells, effects reversed in zinc antiviral protein (ZAP) knockout cells, even though nuclear relocalization of VP remained defective. The demonstrated functional basis for CpG suppression in MVM and potentially other small DNA viruses and the observed intolerance of CpGs in coding sequences, even after codon optimization, has implications for the use of small DNA virus vectors in gene therapy and immunization.

## Introduction

Early investigations into the DNA composition of viruses and their hosts revealed differences in both the relative abundance of each nucleotide and certain dinucleotide combinations [1–3]. This was particularly obvious for CpG and TpA dinucleotides, which were markedly reduced compared to their expected occurrences and their respective counterparts, GpC and ApT. The avoidance of CpG in vertebrate genome DNA was primarily attributed to DNA methylation processes, as methylated CpGs are more prone to missense mutations than their non-methylated counterparts [4, 5]. Supporting this, CpG suppression in genomic DNA sequences is typically most apparent in organisms that highly methylate their genomes (e.g. mammals, other vertebrates), occurs at a reduced frequency in plants with their partial methylation, and is absent from organisms with very low methylation levels (e.g. insects and most other arthropods) [4, 6–9].

It has long been recognized that the dinucleotide bias observed in vertebrate genomes is reflected in RNA viruses infecting them. While almost all single-stranded RNA (ssRNA) viruses, retroviruses and small DNA viruses (<20 kb genome length) show CpG suppression, double-stranded RNA (dsRNA) viruses and large DNA viruses mostly do not [10–12]. This is in contrast to the suppression of UpAs that is present to some extent in the majority of eukaryotes and eukaryotic viruses. UpA composition plays a major in determining rates of turnover of mRNAs through the action of sequence-specific RNAses; there are additionally antiviral pathways, such as RNAseL, which preferentially degrade UpA and UpU rich RNAs in infected cells [13, 14].

While DNA methylation could explain the CpG suppression in DNA viruses and retroviruses, it fails to account for its widespread avoidance in RNA viruses. Indeed, reduced frequencies of CpGs appears to be an adaptive response by the virus to avoid restriction pathways in the cell that recognize CpG-enriched sequences. Indeed, creation of poliovirus or echovirus 7 (E7) mutants with either increased CpG or UpA frequencies in their genomes showed profound attenuation of replication [15–17]; CpG modification reduced the pathogenicity of Influenza A virus and the subsequent immune response towards the virus in a mouse model [18]. Recently, zinc finger antiviral protein (ZAP) was shown to specifically bind to CpG in HIV RNA sequences and attenuate the replication of CpG-high HIV-1 mutants [19]. However, ZAP does not act directly to degrade RNA after binding, and its antiviral effect has recently been shown to depend on the presence of the cytoplasmic protein, KHNYN [20]. We have further shown that ZAP, along with oligoanylate synthetase (OAS) 3/RNAseL, mediates the attenuation of both CpG- and UpA-high mutants of E7 [21].

**Fig. 1.**
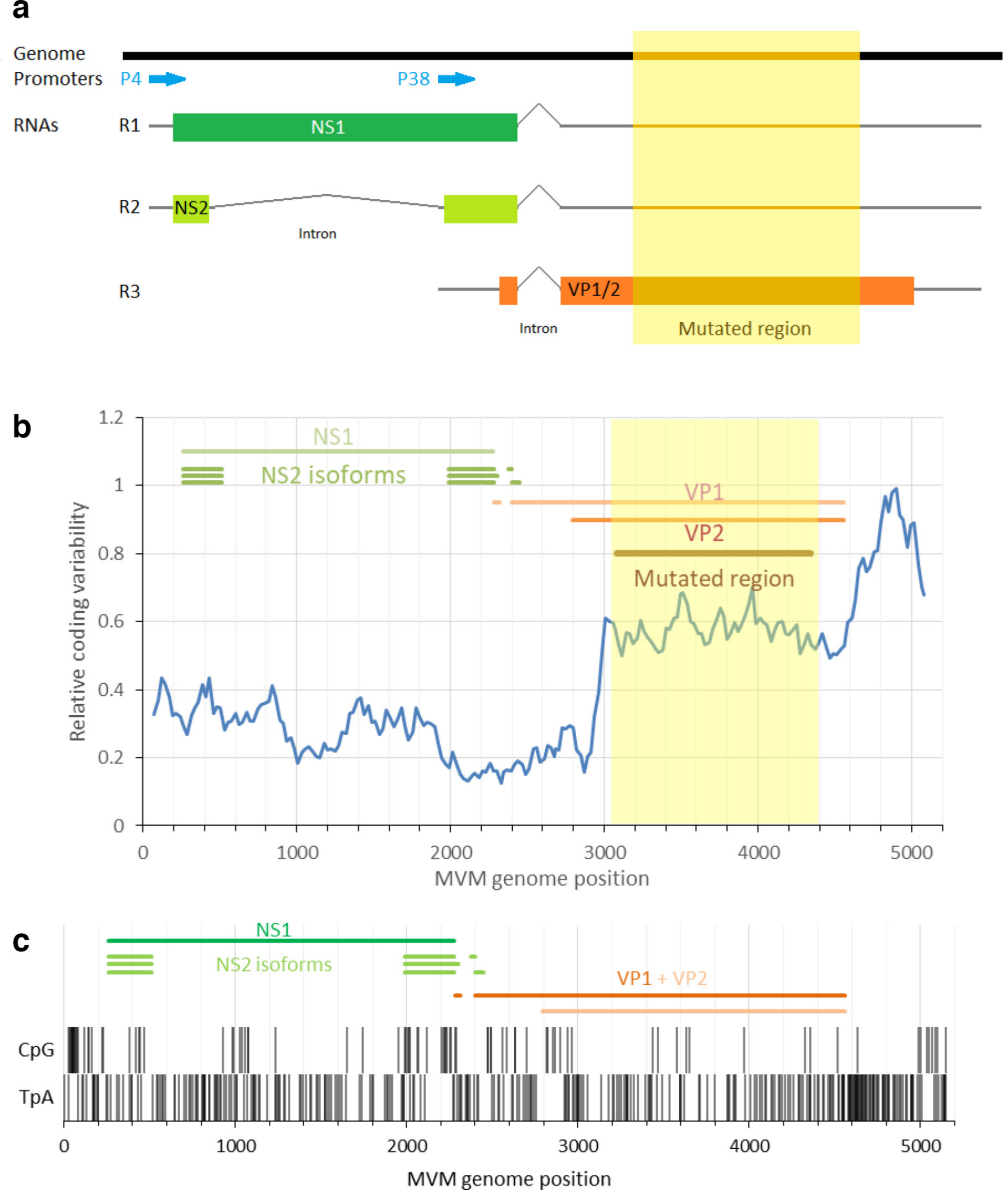
MVM genomic structure and the capsid gene region selected for mutagenesis. (a) Diagram of the MVM genomic structure. Promoters (blue), transcripts (r1-r3) with ORFs (green and orange coloured boxes), mutated region highlighted in yellow. Based on, Fig. 57.10 of Berns and Parrish [65]. (b) SSE was used to calculate the synonymous coding variability observed between MVM and related parvoviruses. The orange region indicates the region chosen for mutations. (c) Distribution of CpGs and TpAs in the MVM genome.

It is currently unknown whether ZAP is also the driving force behind the CpG suppression in small DNA viruses or whether other proteins and mechanisms are involved. To investigate the effects of CpG and TpA compositional modification in small DNA viruses, we developed an *in vitro* cell culture model based on minute virus of mice (MVM), where suppression of both dinucleotides is particularly evident. MVM is an autonomous parvovirus with a single-stranded, linear DNA genome that is 5.1 kb in length and packaged into an icosahedral, non-enveloped capsid with a diameter of 25 nm. The genome encodes two non-structural proteins, NS1 and NS2, driven by the early p4 promoter, and two capsid proteins, VP1 and VP2, whose expression is driven by the later promoter p38 (Fig. 1a) [22]. Capsid proteins are produced and transported into the nucleus, where they assemble into capsids and are packaged with genomic DNA [22]. The central region of the VP structural gene was compositionally modified and used to create mutants from full-length DNAs using an established reverse genetics system [23]. Viruses were assayed for their replication kinetics compared to wild-type (WT) virus; a range of pathway knockout (k/o) cell lines were used to investigate the mechanistic basis for the attenuation of viruses enriched for CpG.

## Methods

### Cell culture, cell lines and reagents

A9 cells [24] and NB324K cells [25] were a kind gift from Peter Tattersall, Yale. Cells were grown in complete Dulbecco’s modified Eagle’s medium (DMEM) [F-12, supplemented with 5% foetal calf serum (FCS) and penicillin/streptomycin)] and incubated at 37 °C with 5% CO_2_. A549 cells, A549 ZAP k/o cells [21], immortalised mouse embryo fibroblast (MEF) cells (a kind gift from Jan Rehwinkel, Oxford) and baby hamster kidney (BHK) cells were grown as above but were supplemented with 10% FCS.

### *In silico* design of codon-optimized, GpC-, CpG- and TpA-modified viruses

SSE version 1.4 [26] (http://www.virus-evolution.org/Downloads/Software/) was used to determine suitable areas for mutation. Synonymous coding variability was calculated using the program SequenceDist within the package. A region with no suppression of synonymous sequences variability within the capsid ORF was chosen, as this was less likely to contain cryptic replication elements or undocumented alternative reading frames. Sequence Mutate in SSE was then used to produce the CDLR sequence and those with altered dinucleotide frequencies based on that region. For the codon-optimized construct (CodOpt), the GeneArt codon optimization tool was used, while Codon Optimization OnLine was used to for the CpG-low codon-optimized mutant (CodOpt CpG-L) [27]. Optimization was performed using codon usage tables for *Mus musculus*. All constructs were synthesized by GeneArt into a commercial plasmid backbone.

### Cloning and plasmid propagation

To generate the mutants, the WT infectious clone pMM984 (a kind gift from David Pintel, Missouri), as well as the insert-containing plasmids, were cut with *Bpu*10I, *Hpa*I and *Xba*I as needed to generate sticky end fragments. These were gel-purified (QIAquick Gel Extraction Kit, Qiagen, UK), ligated with T4 ligase (New England Biosciences UK, UK) and transformed into SURE 2 Supercompetent cells (Agilent, USA) according to the manufacturers’ protocols. Bacterial colonies were grown in Lysogeny broth and plasmid preparations were purified using the HiSpeed Plasmid Midi Kit (Qiagen, Germany) and subsequently used to produce virus stocks. For quantification purposes, the MVM genome was excised out of the pMM984 plasmid with *Bam*HI and gel-purified.

For the R3 subclones, the R3 RNA region of MVM was cloned into a pCAG-loxPSTOPloxP-ZsGreen vector (addgene #51269, a kind gift from Lynn Dustin, Oxford) using *SwaI* and *SacI* restriction sites. The R3 region was amplified and restriction sites were added using two rounds of PCR with a Phusion High Fidelity DNA Polymerase (New England Biosciences, UK) according to the manufacturer’s guidelines (primers: FW1 5′-ATAATTTAAATCACCATTCACGACACCGAAAAGTAC-3′, RV1 5′-ATAGAGCTCTTAGTAAGTATTTCTAGCAACAGG-3′; FW2 5′-GATCGTAACGATAATTTAAATCACC-3′, RV2 5′-GATCGTAACGATAATTTAAATCACC-3′). PCR products and vector were processed as above, but grown in One Shot TOP10 Chemically Competent *Escherichia coli* (Thermo Fisher Scientific, UK).

### Transfection and virus recovery

To produce virus stocks, NB324K cells were transfected using Lipofectamine 2000 (Thermo Fisher Scientific, UK) according to the manufacturer’s recommendations.

One million NB324K cells were transfected with 0.5–2 µg DNA, depending on mutant recovery efficacy. The next day, the cells were split using trypsin and transferred into a T175 tissue culture flask. A non-transfected control was grown in parallel. After 3–5 days, at the onset of cytopathic effect (CPE), cells were harvested by scraping the cells into the culture medium. Cells and medium were spun down and separated. The cell pellet was resuspended in 3 ml vTE (50 mM Tris-hydrochloride, 0.5 mM EDTA, pH 8.7 [28]) and freeze-thawed three times. The cell lysate was spun down (1500 r.p.m., 20 min, 4 °C) and the cleared lysate was sterile-filtered, aliquoted, frozen at −80 °C and titrated.

### Virus titration

TCID_50_s were used to quantify virus concentrations. Ninety microlitres of NB324K cell suspension containing 3000 cells was mixed with 10 µl viral suspension (serial 10-fold dilutions in complete DMEM) in a 96-well plate. Ten days post-infection, the TCID_50_ score was determined using the Reed–Muench method [29].

### Virus infections

Cells were seeded overnight before being infected. Plate sizes were chosen in accordance with individual experiment needs. Cell numbers were chosen according to experiment length, seeding at roughly 10% confluence for experiments with total incubation periods of 48 h or longer, and seeding at roughly 25% confluence for experiments with a total incubation period of 24 h or less. Cells were infected with either equal TCID_50_ units or equal copy numbers. Viral stocks were diluted in Opti-MEM. Cell supernatant was replaced by the viral suspension and incubated for 1 h at 37 °C, while shaking the plate occasionally. Afterwards, the cells were washed 3× with phosphate-buffered saline (PBS) and fresh DMEM with 5 or 10% FCS added as appropriate.

### Serial passage

A9 cells were cultured in 25 cm^2^ tissue culture flasks and were infected with virus stocks [multiplicity of infection (m.o.i.) of 0.5]. For the serial passage, viral supernatant was added to fresh A9 cells twice a week in a dilution that would allow complete CPE within 3–4 days (WT: 1 : 50; TpA-H: 1 : 25; CpG-H half-mutants: 1 : 2).

### *In vitro* transcription and translation

Plasmids were linearized using *Apa*LI for 4 h at 37 °C and purified (QIAquickPCR Purification Kit, Qiagen, UK). One microgram of of DNA was used to transcribe capped and poly-adenylated messenger RNAs using the mMESSAGE mMACHINE T7 Transcription Kit (Thermo Fisher Scientific, UK) according to the manufacturer’s description and purified using the RNA Clean and Concentrator-25 Kit (Zymo Research, USA). RNA integrity was determined via the Bioanalyser, using the RNA 6000 Pico Kit (Agilent, USA). Two micrograms of RNA were used in the Rabbit Reticulocyte Lysate System, Nuclease Treated (Promega, UK) according to the manufacturer’s instructions. Samples were then immunoblotted.

### Transcription experiments

Cells were seeded and used for transfections the next day. Cells were transfected with 500 ng DNA per 100 000 cells using Lipofectamine 2000 (Thermo Fisher Scientific, UK) according to the manufacturer’s guidelines. Four hours post-transfection, 5 µg ml^−1^ Aphidicolin (Santa Cruz Biotechnology, USA) was added.

### TLR9 stimulation and inhibition

For TLR9 stimulation, cells were stimulated with 1 µg ml^−1^ purified *E. coli* DNA (InvivoGen, UK) for 18 h, with or without 2 h pre-treatment with 5 µM ODN2088 (InvivoGen, UK). For TLR9 inhibition, cells were pretreated with 5 µM ODN2088 for 2 h, before being infected with 1000 virus genomes/cell as described above.

### DNA and RNA extractions

Viral stocks were pretreated with DNAseI (Roche, Switzerland) prior to DNA extraction. Then 0.1 mg ml^−1^ DNase and 5 mM MgCl_2_ was added to the samples, which were incubated at 25 °C for 25 min. Control (no-virus) lysate was spiked with 10^9^ *Bam*HI-excised MVM copies to evaluate DNAse digestion and DNA recovery rate. DNA was extracted and purified with either the DNeasy Blood and Tissue Kit (Qiagen, UK) or the Quick-DNA Viral Kit (Zymo Research, USA) according to the manufacturers’ guidelines. RNA was extracted and purified using the RNAeasy Mini Kit (Qiagen, UK) according to the manufacturer’s guidelines. DNA and RNA concentration was then measured using the Qubit (Thermo Fisher Scientific, UK).

### Quantitative real-time PCR

For the quantitative real-time PCR, the QuantiFast SYBR Green RT-PCR Kit (RNA) or QuantiFast SYBR Green PCR Kit (DNA) (Qiagen, UK) were used according to the manufacturer’s instructions, loading 80 ng RNA or DNA per well. For viral DNA quantification, primers in the NS region were used (FW: 5′-AGTTTGCCATGCTATTTGC-3′ and RV: 5′-AATCTGCTTGCTGCCTTT-3′, from Zhan *et al.* [30]) with a standard curve derived from serially diluted *Bam*HI-excised MVMp. For viral RNA quantification, primers targeting MVM R1 (FW: 5′- CCAACTCCTATAAATTTACTAG −3′, RV: 5′-ACCAAGTGATTTCAGGCCTTAG-3′, from Li *et al.* [31]) or R3 (FW: CACCATTCACGACACCGAAAAG, same RV as R1) were used. For IL-1β, FW: 5′-GCAACTGTTCCTGAACTCAACT-3′and RV: 5′-ATCTTTTGGGGTCCGTCAACT-3′ were used [32]. Samples were normalized to the species-appropriate GAPDH (human FW: 5′-GAAATCCCATCACCATCTTCCAGG-3′, RV: 5′-GAGCCCCAGCCTTCTCCATG-3′, from Ahn *et al.* [33]; mouse FW: 5′- CGACTTCAACAGCAACTCCCACTCTTCC −3′, FW: 5′- TGGGTGGTCCAGGGTTTCTTACTCCTT −3′, from Banerjee *et al.* [34]) using the ΔΔCT method [35].

### Miseq sequencing

Purified DNA underwent second-strand synthesis using Sequenase Version 2.0 (Thermo Fisher, UK) and random primer 9 (New England BioLabs, UK). Libraries were prepared from 1 ng of the resulting double-stranded DNA using the Nextera XT kit (Illumina, UK) and sequenced on the MiSeq platform with reagent kit v2 (500 cycles). After adapter removal and quality trimming using Trimmomatic [36], reads were aligned against the reference sequence (GenBank no. J02275) and the derived mutant sequences using Bowtie 2 [37].

### Immunofluorescence (IF)

Cells were seeded into cellview cell culture dishes (VWR, USA) or Falcon 96-well imaging microplates (Corning, USA). The next day, cells were infected with 1000 genome copies per cell. After 18 h incubation, the cells were submerged into 4% paraformaldehyde for 10 min, neutralized with 0.1 M glycine for 5 min and permeabilized in 0.1% Triton in PBS for 20 min. The cells were then blocked with 2% FCS in PBS for 20 min, washed 3× with PBS and stained for 1 h with the primary antibodies (Mab3 [38]: 1 : 100, monoclonal mouse antibody; NS1/2 [39]: 1 : 400, polyclonal rabbit antibody, both kind gifts from Peter Tattersall). Subsequently, samples were washed 3× with PBS and stained with the secondary antibodies (1 : 2000 anti-rabbit Alexa Fluor 488 and either 1 : 2000 anti-mouse Alexa Fluor 594 or 647; all from Thermo Fisher Scientific, UK), stained with Hoechst 33342 (1 : 2000, Thermo Fisher Scientific, UK) for 10 min, and washed 3× in PBS. Slides were analysed using a Leica DMIRE2 microscope and Q capture pro 7 software or an Olympus IX73 fluorescence microscope and the accompanying software. Post-acquisition analysis was conducted using Imaris (Bitplane) or Fiji (v1.49) software [40].

### Immunoblotting

Cells were infected with 1000 viral copies/cell for 18 h, washed with PBS, scraped off the cell culture dish and resuspended in Laemmli buffer. *In vitro* translation products were diluted with 4× Laemmli buffer (Bio-rad, UK). Samples were incubated for 5 min at 95 °C and spun down. Lysate was loaded onto a 10-well (10 µl) or 15-well (5 µl) 4–15% gradient mini protean TGX SDS-PAGE gel (Bio-rad, UK) and run for 4 h at 40 V. Gels were then transferred using the Trans-blot Turbo transfer system (Bio-rad, UK) according to the manufacturer’s guidelines. Membranes were blocked in 5% skimmed milk powder in 0.1% PBS-T, incubated ON at 4 °C with primary antibodies (Tatt3 [38]: 1 : 10000; NS1/2 [39]: 1 : 10000, both polyclonal rabbit antibodies and kind gifts from Peter Tattersall; GAPDH (ab70699, abcam, UK)). Membranes were washed 3× in 0.1% PBS-T and incubated for 1 h at 25 °C with goat anti-rabbit IgG H and L HRP antibody (ab205718, abcam, UK). Membranes were washed 3× in 0.1% PBS-T, substrate was added (Amersham ECL Prime Western Blotting Detection Agent, GE Lifesciences) and images were exposed using the ImageQuant LAS4000 (GE Healthcare, UK). Images were analysed using ImageJ. The background was digitally subtracted and bands were quantified by measuring the mean grey value over equal areas.

## Results

### Suppression of CpG and TpA dinucleotides in parvoviruses

**Fig. 2.**
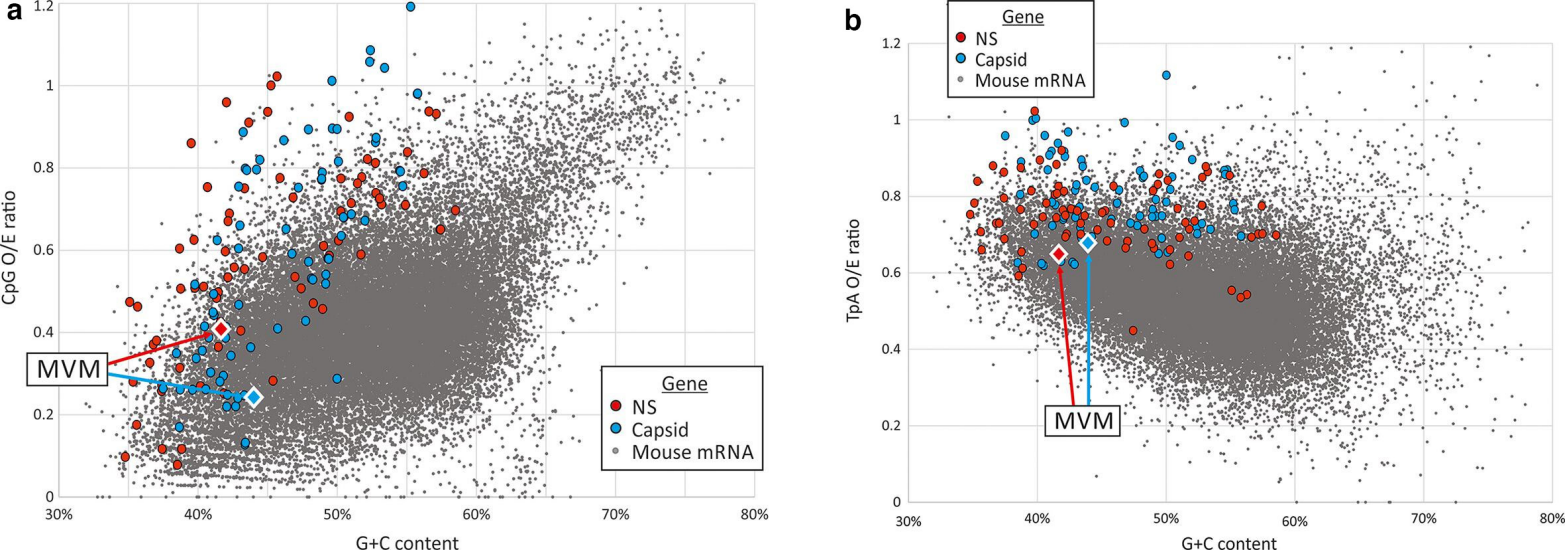
CpG and TpA dinucleotide distribution in parvovirus genes. Comparison of frequencies of CpG and TpA dinucleotides in MVM NS- and capsid-encoding genes (red and blue filled diamonds) and representative other parvoviruses (circles) with those of mouse (*Mus musculus*) mRNA sequences (each grey point represents a separate gene). Observed (o) CpG and TpA frequencies were normalized to values expected (e) from their G+C content.

Representative sequences of autonomous parvoviruses infecting mammals were divided into non-structural (NS) and VP1 (minor-capsid) coding regions and their mononucleotide and dinucleotide compositions were determined using the program Composition Scan in the SSE v1.3 package [26] and compared with those of mouse cellular mRNA genes (Fig. 2a, b). Both NS and VP1 genes of parvoviruses showed varying degrees of CpG suppression (Fig. 2a), with ratios of observed frequencies to expected frequencies based on G+C content ranging from <0.1 to 1.2 (*y*-axis). Suppression was greatest for parvovirus genomes with lower G+C content, a relationship recapitulated to an extent in mouse mRNA sequences. Frequencies of TpA dinucleotides in NS and VP1 genes showed a lower degree of suppression (Fig. 2b), again following a relationship observed in human mRNA with lower TpA frequencies in sequences with higher G+C content. The highlighted gene sequences of MVM show degrees of CpG and TpA suppression that are typical of other parvoviruses with low G+C content. CpG frequencies were 0.41 and 0.25 for the NS and VP1 genes, respectively, while TpA was 0.65 and 0.69. A graphical representation of the positions of CpG and TpA dinucleotides is shown in Fig. 1c.

### Effect of dinucleotide frequency modification on the replicative fitness of MVM

To study the mechanism or selection pressure underlying the suppression of CpG and TpA dinucleotides in MVM, the frequencies of CpG and TpA were modified in a region within the VP gene using synthetic DNA sequence inserts. This region was chosen as it contains a single open reading frame and has no known structural features or regulatory elements (Fig. 1a). This is supported by a high synonymous sequence variability, indicating conservation at the protein level but not the DNA or RNA level (Fig. 1b).

The native sequence in the chosen region (24.4% of the entire genome) was replaced by synthetically generated sequences containing a range of synonymous changes that left protein coding intact. The CDLR sequence was used as a mutagenesis control – codon order was scrambled to disrupt any underlying DNA or RNA structural elements or alternative reading frames in the sequence, while keeping coding, codon usage and dinucleotide frequencies identical to those of the WT sequence (Table 1). The CpG-high (CpG-H) mutant possessed maximized frequencies of CpG dinucleotides while retaining protein coding, achieving a ratio of 1.62× the expected frequency of CpGs based on this G+C content (O/E ratio; Table 1), 13× that of the native MVM sequence. TpA-high (TpA-H) sequences were similarly maximized to 1.23× the expected frequencies, approximately double that of the WT sequence.

**Table 1.** Dinucleotide composition of MVM mutant regions

Mutant	Fragment length	G+C content	CpG		GpC		TpA	
			No.	O/E* ratio	No.	O/E ratio	No.	O/E ratio
WT	1257	42.32 %	7	0.126	58	1.04	59	0.585
CDLR	1257	42.32 %	7	0.126	58	1.04	59	0.585
CpG-H	1257	47.89 %	**117†**	**1.634**	70	0.978	59	0.716
CpG-H first half	676	46.75 %	**59**	**1.612**	39	1.066	37	0.8
CpG-H second half	582	49.14 %	**58**	**1.66**	31	0.887	22	0.606
CpG-H 42% G+C	1257	42.32 %	**78**	**1.398**	58	1.039	60	0.595
TpA-H	1257	42.32 %	7	0.126	59	1.058	**124**	**1.227**
GpC-H	1257	42.32 %	7	0.125	**91**	**1.626**	59	0.585
G+C-Max	1257	**57.44 %**	7	0.068	88	0.858	59	1.061
CodOpt	1257	**61.10 %**	**70**	**0.611**	108	0.943	23	0.524
CodOpt CpG-L	1257	**57.04 %**	7	0.069	92	0.908	25	0.444
								
Whole genome	5149	42.12%	86	0.378	256	1.1251	312	0.7428

*O/E: observed over expected.

†Deliberately or secondarily modified frequencies are indicated in bold in a shaded cell.

To generate infectious virus, DNA clones of WT and mutated MVM genome sequences were transfected into NB324K cells and cell lysate was harvested at the onset of cytopathic effects (3–5 days post-transfection) and passaged for further replication in NB324K cells. Cell lysate from this passage was collected, aliquoted and titred, and used as a source of virus for phenotypic characterization. Viruses were titred in NB324K cells to estimate TCID_50_s, while DNA concentrations in virus stocks were measured by qPCR using MVM-specific primers in the non-mutated NS region. Virus stocks of WT and mutant MVM virus stocks showed similar genome copy numbers, ranging between 10^10^ to 10^11^ DNA copies ml^−1^.

**Fig. 3.**
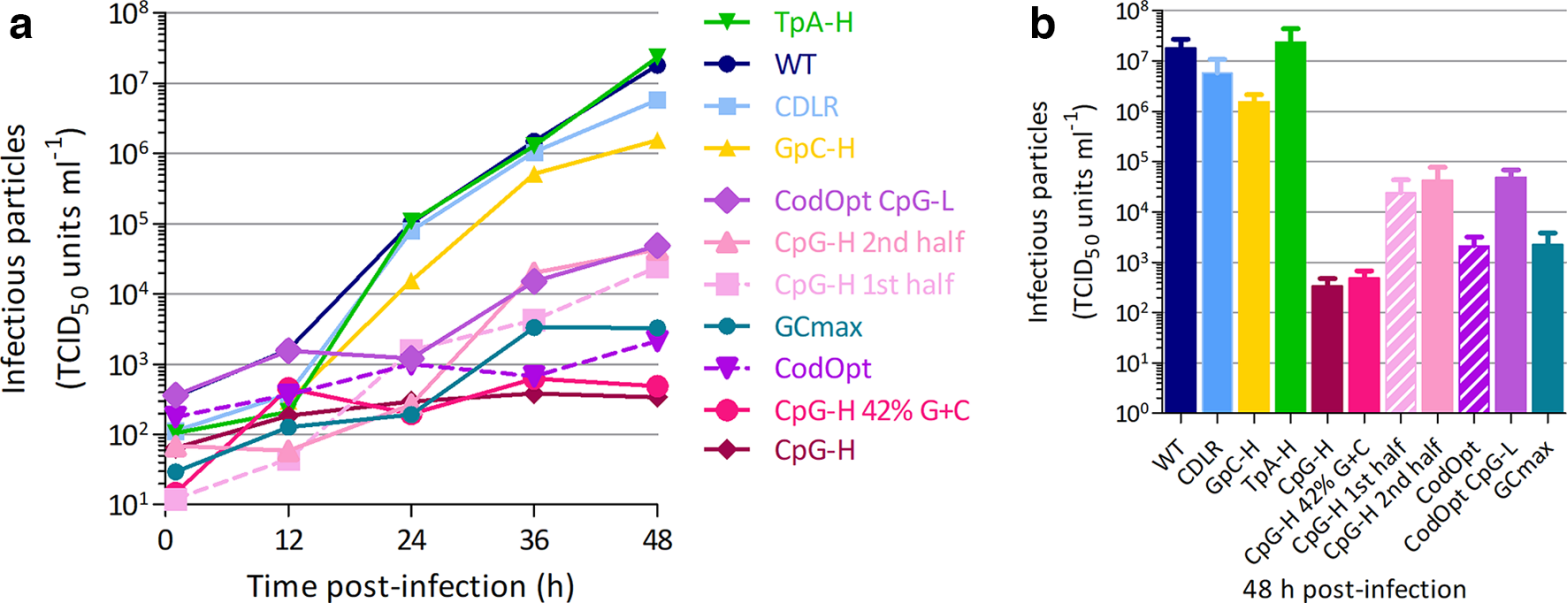
Effects of V2 sequence modifications on MVM replication kinetics. (a) Multi-step growth curve in the A9 cell line infected with 1 TCID_50_ unit/cell. Supernatant was harvested at different time points and titred in NB324K cells. Data points represent the mean of three biological replicates. (b) Cross-section of the growth curve at 48 h. Bar heights represent the mean of three biological replicates; error bar show sems.

Several MVM mutants showed substantial differences in replication kinetics from the WT virus in both in A9 and NB324K cell lines. These were apparent over the time course of a standard replication assay (Figs 3a and S1, available in the online version of this article) and in a cross-sectional analysis of virus titres in cultures in the A9 cell line at 48 h (Fig. 3b). The replication kinetics of the essential CDLR control was comparable to that of the WT virus in both cell lines, indicating that replication defects observed in other mutants (described below) did not originate from any DNA/RNA structure disruption caused by CDLR scrambling of the underlying coding sequence, or through the existence of uncharacterized alternative open reading frames.

However, modification of CpG frequencies led to a profound replication defect of MVM. The CpG-high mutant containing 117 CpG nucleotides instead of 7 barely replicated, with little net increase in supernatant infectivity titres over the duration of the assay. To investigate the dose-dependence of CpG addition on replication kinetics, a pair of mutants were generated in which the CpG modification was made either side of the *Hpa*I restriction site in the middle of the mutated region, with 59 in the 5′ half and 58 in the 3′ half (Table 1). Both CpG-high half-mutants were substantially attenuated but to a lesser extent than the full-region CpG-high mutant (containing 117 CpGs), with both showing an approximately three log reduction in replication abilities in A9 cells compared to WT. However, in contrast to the marked attenuation effects of increasing UpA sequences on the replication of E7 and influenza A virus [18, 21], the replication of the TpA MVM mutant matched that of the WT virus.

As controls we constructed a series of further mutants to investigate potential compounding effects of sequence manipulations on replication phenotypes. Firstly, increasing CpG frequencies potentially leads to a greater degree of DNA secondary structure in genomic DNA, or in RNA expressed in MVM structural gene mRNAs. To investigate this possibility separately from CpG frequency changes, we constructed a GpC-H mutant in which the similarly self-complementary GpC dinucleotides were increased in frequency (Table 1). However, the GpC-H mutant (with 91 GpC dinucleotides) replicated similarly to WT, providing no evidence that the attenuation of the CpG-H mutant originated from structural changes in the RNA. As a further control, we recognize that a further side-effect of increasing CpG content was to add further C and G residues to the sequence; in the VP2 mutated region this led to an increase in G+C content from 42.3–47.9%. To verify that the replication defect of the CpG-H mutant was not the result of the G+C content change, we constructed a new mutant in which the inserted C and G bases were compensated by removal elsewhere so that overall G+C content could be preserved. While retaining mononucleotide base composition limited the number of CpG dinucleotides that could be added (71 instead of 110), the resulting mutant (CpG-H 42% G+C) was highly attenuated (Fig. 3), indicating that perturbation in G+C content was unlikely to be the cause of attenuation in the CpG-H mutant.

### Effect of codon optimization on MVM replication

Codon optimization is a frequently used mutational step to enhance expression of proteins from viral and vector-expressed mRNAs in biotechnology and vaccine applications. To investigate the effects of codon optimization on MVM replication, the insert region of VP2 was codon-optimized by a standard algorithm (see the Methods section) that is designed to maximize translation of the encoded protein (CodOpt). Remarkably, replication of the codon-optimized mutant was almost as attenuated as the CpG-high mutant, despite its VP region coding sequence being modified in a way that would conventionally enhance translation. Potentially, it was the addition of CpG dinucleotides to this genome region (7 to 70 and respective O/E ratios of 0.13 and 0.61; Table 1), rather than translation effects that led to its observed attenuation.

To investigate this potential compounding factor, we created a further mutant in which the MVM V2 coding region sequence was codon-optimized but the number of CpG dinucleotides was maintained at WT level (CodOpt CpG-L). This mutant, however, also showed attenuation (approximately 2.5 logs lower than WT), although to a lesser extent than the four log reduction in the original CodOpt mutant. The other effect of codon optimization was to increase G+C content from 42.3 (WT) to 61.1 and 57.0% in the CodOpt and CodOpt CpG-L mutants, respectively. This may have alternatively influenced replication independently of codon usage and in a non-CpG-dependent manner. To investigate this, we constructed a further mutant in which G+C content was maximized (57.4 %) while retaining native frequencies of CpG and TpA dinucleotides (GCmax; Table 1). This mutant showed comparable reduction in replication to that of the CodOpt mutant (approximately four logs; Fig. 3b), providing evidence that large-scale modification of genomic G+C composition does indeed have a substantial effect on replication separate from CpG representation.

### Immunofluorescence assay and analysis

**Fig. 4.**
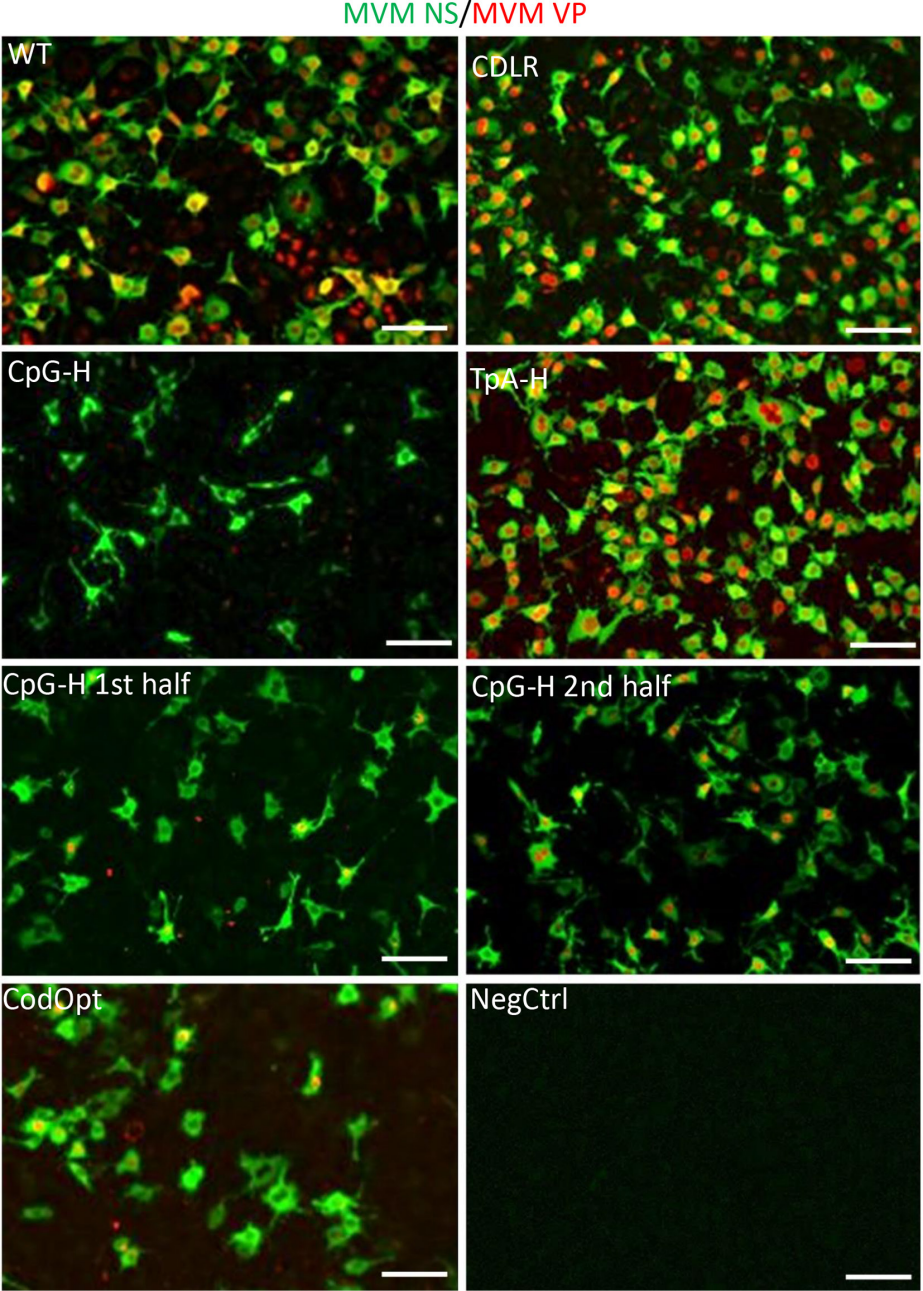
Cellular distribution of MVM viral proteins in A9 cells. Immunofluorescence staining of viral NS (green) and VP (red) proteins at 18 h post-infection with 1000 viral copies/cell. Uninfected cells were used as a negative control. *n*=3, scale bar 100 µm, representative image shown.

To compare the spatial and temporal distribution of gene expression from the NS and structural genes and how this was influenced by dinucleotide frequency modifications, infected cells were fixed with paraformaldehyde 18 h post-infection with WT and mutants of MVM and stained for the viral NS and VP proteins using specific antibodies (Figs 4 and S2). To ensure that cells were exposed to equal amounts of viral particles, cells were infected with equal copy numbers of WT and mutant virus stocks based on DNA/infectivity measurements (Fig. S3). Cells infected with WT and TpA-mutants of MVM showed similar frequencies and fluorescent intensities of NS and VP expression, while there was small reduction in the CDLR mutant. Contrastingly, the CpG-high and codon-optimized mutants showed greatly reduced frequencies and expression levels, particularly of the VP gene that contains the region modified by mutagenesis.

**Fig. 5.**
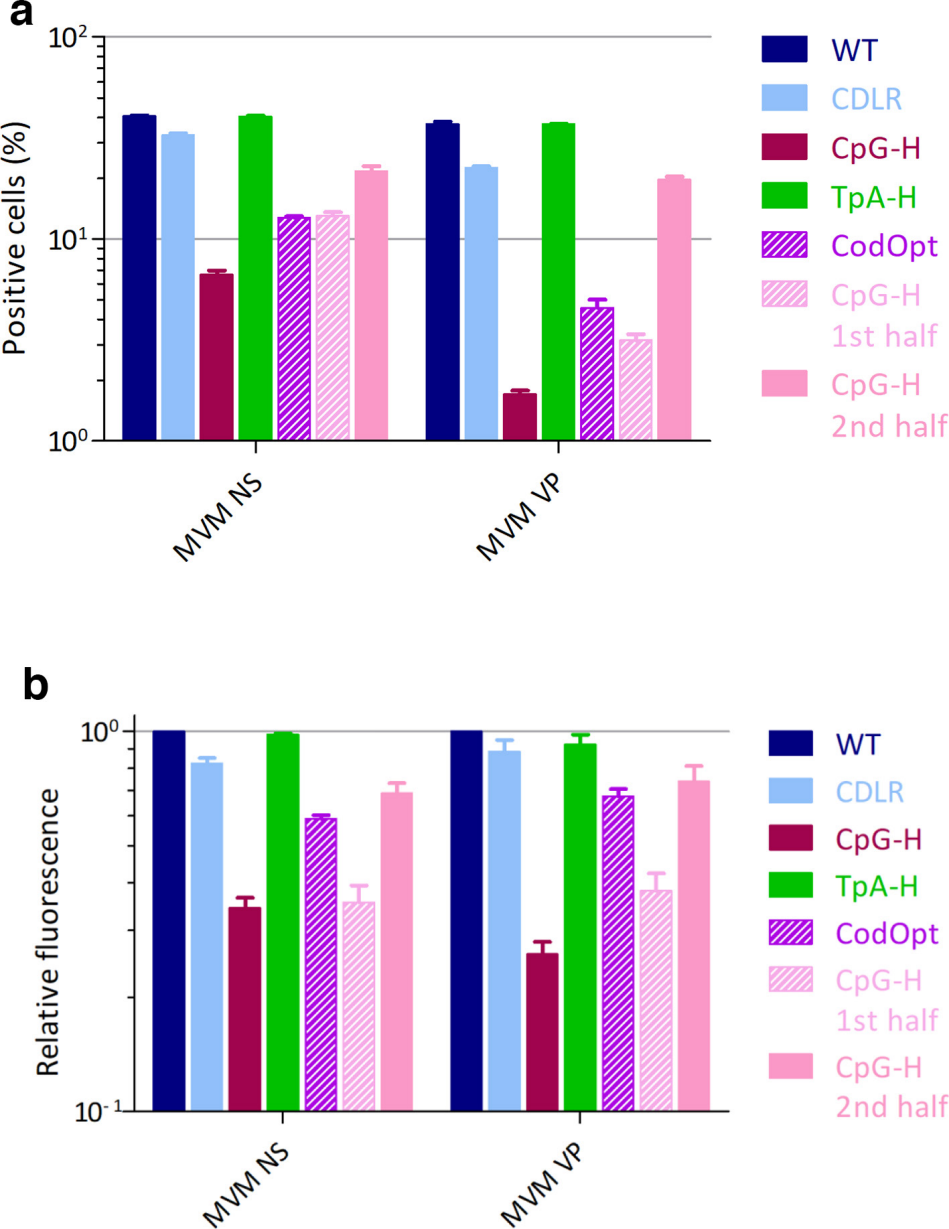
Quantitative analysis of viral protein expression. Quantification of immunofluorescence staining of viral NS and VP proteins using Imaris and Fiji at 18 h post-infection with 1000 viral copies/cell. Uninfected cells were used as a negative control. *n*=3, +/−sem. (a) Percentage of cells expressing NS or VP. (b) Relative fluorescence intensity of NS or VP compared to WT MVM.

**Fig. 6.**
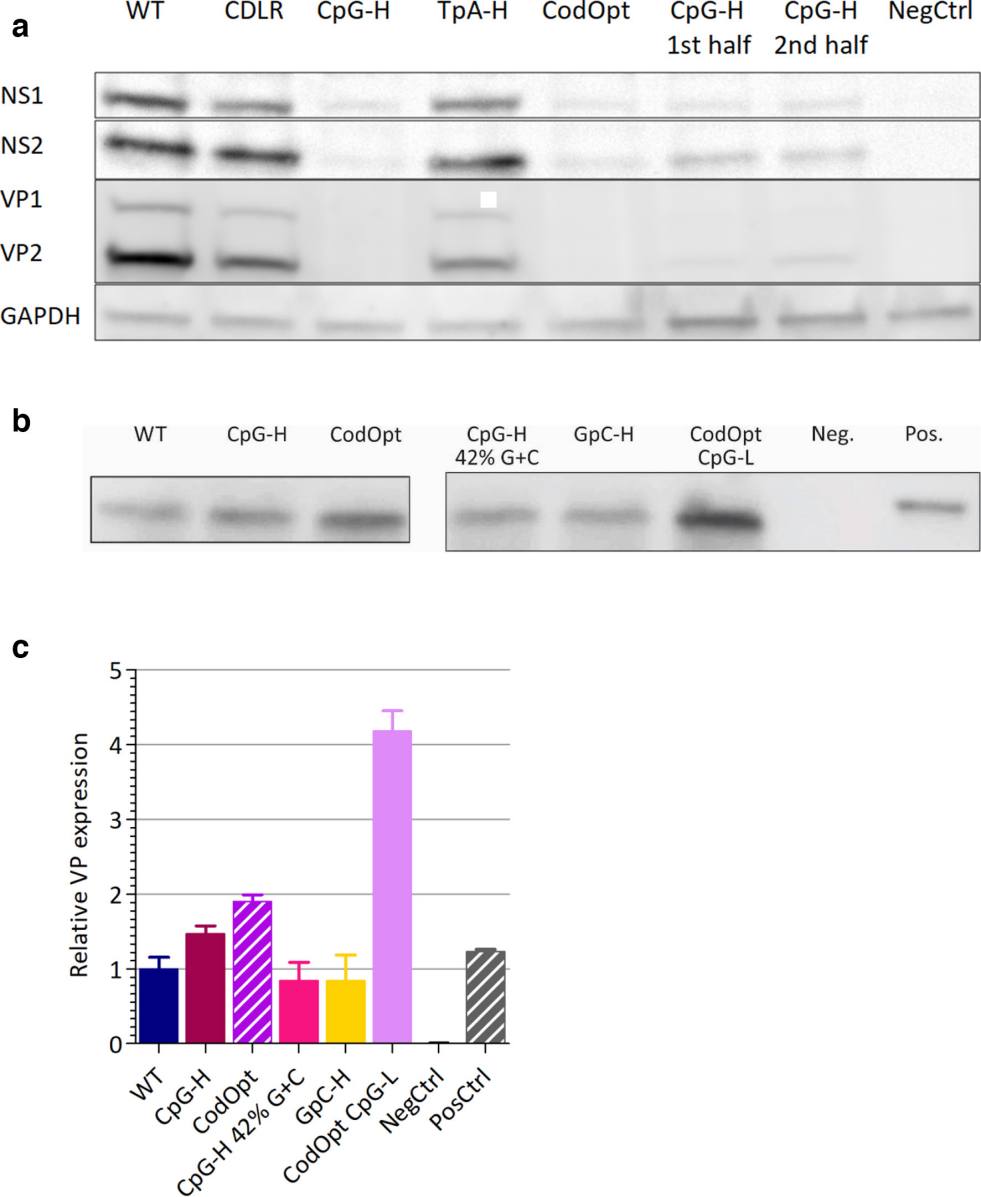
MVM gene expression and translation. (a) Viral protein expression 18 h post-infection with 1000 viral copies/cell. Uninfected cells were used as a negative control. *n*=3, representative image shown. (b) *In vitro* translation of a truncated VP2. RNA was transcribed into mRNA *in vitro* via a T7 promoter using the synthetic plasmids containing the different MVM inserts. Equal amounts of the RNA were then translated using an *in vitro* rabbit reticulocyte and measured using immunoblotting. The negative control is a mock translation with no RNA added, while the positive control is an infected cell lysate (VP2 band shown). (c) Quantification of VP2 protein levels (from Fig. 6b) by ImageJ expressed as the ratio to that of the WT gene (*y*-axis). Bar heights shows the mean of three replicates; error bars show +/−sem.

To quantify these reductions, representative areas of immunostaining from each mutant were analysed and the numbers of fluorescent foci and the intensity were determined in each section (Fig. 5a, b). This analysis showed only minor differences in frequencies and fluorescent intensity in expressing cells between WT, CDLR and TpA-high mutants. In contrast, it quantified a profound reduction in expressing cell frequencies and intensities in CpG-high and codon-optimized mutants. However, while the numbers of VP-expressing cells were lower than those expressing the NS protein, they both showed a comparable reduction in fluorescent intensity relative to WT in the cells that were infected. To separately verify these results using a different virus detection method, cells were infected for 18 h and viral protein content was measured using Western blot (Fig. 6a). Similarly to the immunofluorescence images, CDLR and TpA-H mutants showed only slight reductions in protein levels compared to WT, in contrast to the much greater reduction for the CpG-high and codon-optimized RNA.

### RNA and protein expression levels

To investigate whether the observed differences in protein expression between WT virus and compositionally modified mutants was mediated at the translational or mRNA level, the translation rates of native and modified VP sequences were first determined in an extra-cellular *in vitro* translation assay. This assay determines translation efficiency independently of other potential virus-attenuating effects of altered composition mediated through mRNA stability or virus replication. A synthetic plasmid containing the partial VP sequence used for cloning (spanning positions 2647–4347 of the MVM genome) was linearized and capped RNA transcripts were synthesized. One microgram of each RNA was used in an *in vitro* rabbit reticulocyte translation assay and protein levels were assayed at 1.5 h using Western blot (Fig. 6b, c). In contrast to their marked effects on virus replication, the translation rates of CpG-H and CodOpt VP sequences were similar relative to that of the WT sequence. These observations provide no evidence that differences in the translation efficiency of the capsid gene influenced the attenuated phenotype of CpG-high mutants.

**Fig. 7.**
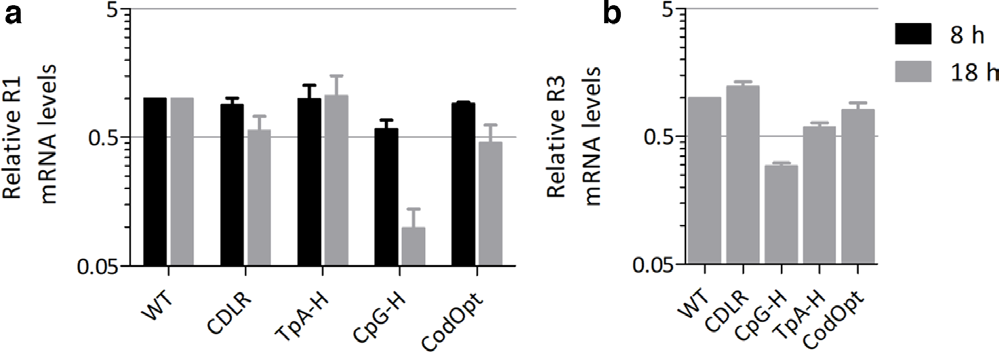
MVM R1 mRNA levels post-transfection. MVM R1 mRNA expression 8 and 18 h post-transfection normalized to cellular GAPDH and WT R1 levels. *n*=3, +/−sem. (a) Plasmid pMM984, viral infectious clone, replication inhibited by Aphidicolin. (b) Plasmid pCAG_MVMp_R3, expression vector containing MVM R3 subclone.

As an alternative mechanism of attenuation, mRNA sequences with high CpG frequencies may be unstable or more readily degraded in the cytoplasm and therefore disrupt MVM replication. To evaluate this possibility, mRNA levels of the full-length genomic transcripts were quantified in cells transfected with MVM DNA in the presence of a replication inhibitor (Aphidicolin) to avoid compounding the effects of virus replication differences on the measured outcomes. Because of the expression strategy of MVM, it was not possible to independently compare mRNA levels of NS- and VP1-encoding mRNAs, since the mRNA for NS contains the same modified sequences downstream from the NS gene as the VP1 gene (Fig. 1a). Viral RNA transcripts were quantified using primers targeting the NS1 region (R1) after 8 and 18 h (Fig. 7a). While the RNA levels at 8 h were initially similar, a reduction of R1 RNA levels relative to those of the WT and CDLR viruses was observed in the CpG-H mutant and to a lesser extent in the codon-optimized mutant at 18 h post-transfection. To further isolate the effects of virus replication on RNA expression, we constructed a sub-viral (R3) expression system in which WT and compositionally modified mutants of MVM cDNA sequences encoding the MVM VP proteins were cloned into a pCAG expression vector with transcription driven by the CAG promoter [41, 42]. This avoided possible compounding effects of MVM-related replication processes, such as expression of NS genes, on VP gene stability or turnover. Only the CpG-high mRNA showed a reduction in expression, 3-fold compared to the >10-fold reduction in the corresponding virus-expressed transcripts (Fig. 7b). The levels of the codon-optimized and UpA-high mRNAs were comparable to those of the WT and CDLR. These findings are therefore consistent with a role for RNA stability differences of CpG-modified sequences in their observed viral attenuation.

### Stability of compositionally modified mutants

The attenuation of CpG-high mutants of MVM creates a selection pressure for the reversion of introduced CpG dinucleotides into the mutated VP region. To investigate the stability of the two CpG-high half-mutants along with the non-attenuated TpA-H and WT viruses as controls, viruses were serially passaged at high m.o.i. for 11 passages over a period of 5 weeks. Each passage was terminated on development of a full CPE. At the end of the passaging, viral genomic DNA was extracted and subjected to next-generation sequencing to investigate the occurrence of nucleotide (and inferred amino acid) substitutions in both mutated and non-mutated regions of the genome (Fig. S4). The CpG-H first and second half-mutants were remarkably stable on cell culture passage, with the CpG-H first half-mutant showing no reversions in the CpG-enriched mutated regions, and the occurrence of minor populations in the non-mutated NS region. The CpG-H second half-mutant showed two partial CpG reversions in the mutated region, and four partial or complete substitutions in the non-mutated first half-region. It also displayed a partial TpA reversion in the NS region. The TpA-H and WT viruses showed single, partial changes in the VP gene region that had no effect on CpG or TpA representation. In addition to demonstrating the high genetic stability of the MVM mutant on long-term *in vitro* passage, nucleotide sequencing provided no evidence for any CpG-induced amino acid changes, deletions, insertions or other genome defects in either coding or replication regions that might underlie their substantial attenuation.

### Mechanism of CpG-mediated attenuation of MVM replication

**Fig. 8.**
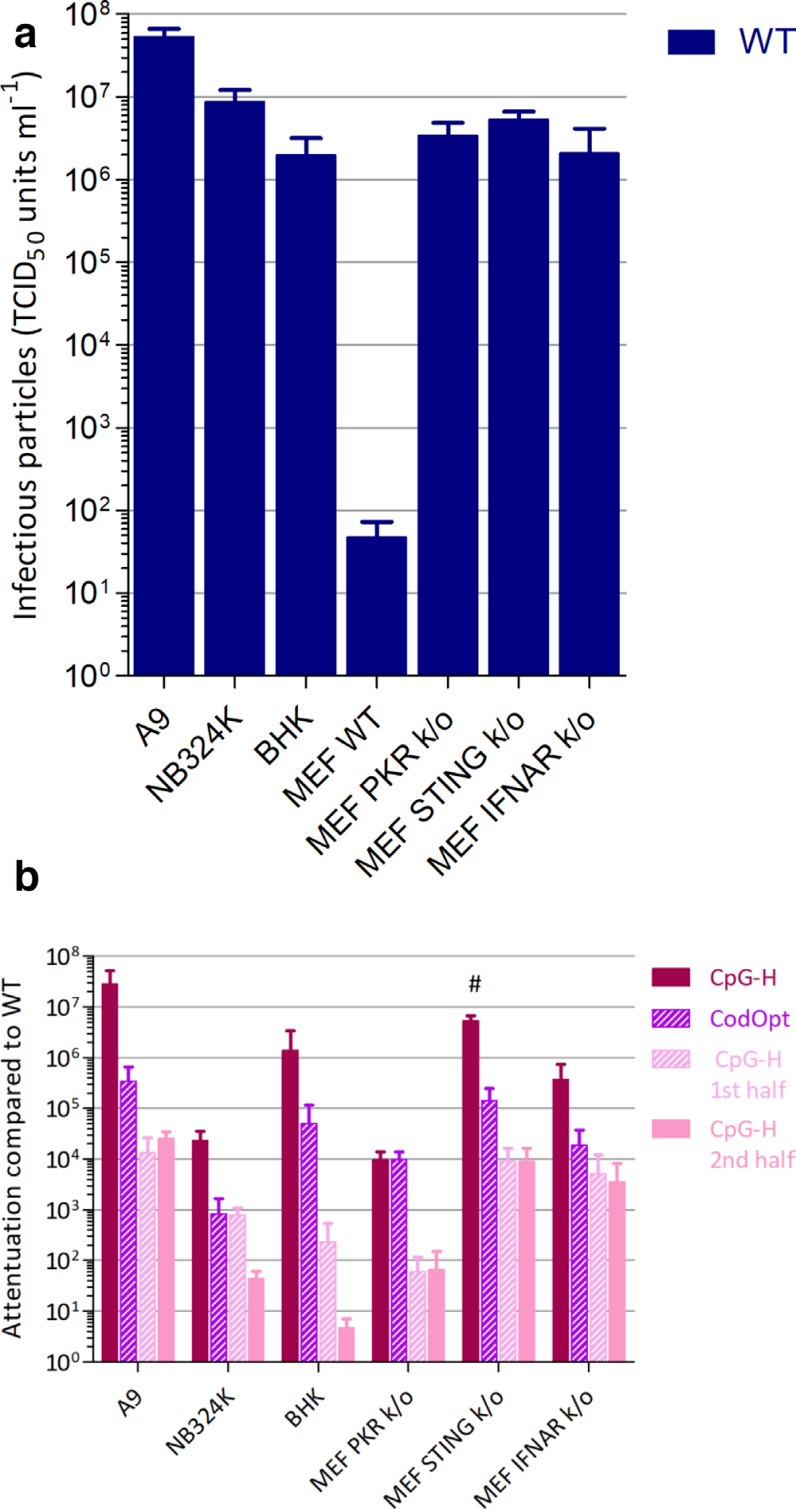
Cell line-associated differences in MVM replication. Infectivity of MVM 72 h post-infection (1 TCID unit/cell) in different cell lines. *n*=2, +/−sd. (a) Viral titres of MVM WT virus. (b) Attenuation of CpG-high and codon-optimized mutants of compared to WT. # indicates samples where no virus could be recovered, thus underestimating attenuation.

Differences in the degree of attenuation of CpG-high and codon-optimized mutants of MVM were apparent from their different replication kinetics in A9 and NB324K cell lines (Fig. 3 and S1). To investigate the extent to which attenuation was influenced by cell type, the susceptibility of a range of cell lines was evaluated through infection by WT virus at an m.o.i. of 1 and assay of supernatant infectivity at 72 h (Fig. 8a). NB324K, BHK and several immortalized mouse embryo fibroblast (MEF) cell lines with CRISPR-targeted knockout of different innate immune signalling pathway or effector genes were susceptible to MVM and could be evaluated for CpG-mediated attenuation (Fig. 8b; raw replication data are shown in Fig. S5).

The replication levels and degrees of attenuation of the CpG-H, CodOpt and both CpG-H half mutants were comparable in the NB324K and BHK cell lines. While the replication of MVM in unmodified MEF cells could barely be detected, knockout of protein kinase R (PKR), stimulator of interferon genes (STING) and interferon α receptor (IFNAR) reversed this restriction and enabled MVM replication to occur at comparable levels to that in A9 cells. However, in each k/o cell line, CpG-mediated attenuation was comparable to that observed in A9 cells, providing no evidence that these pathways participated in its attenuated phenotype compared to WT virus.

**Fig. 9.**
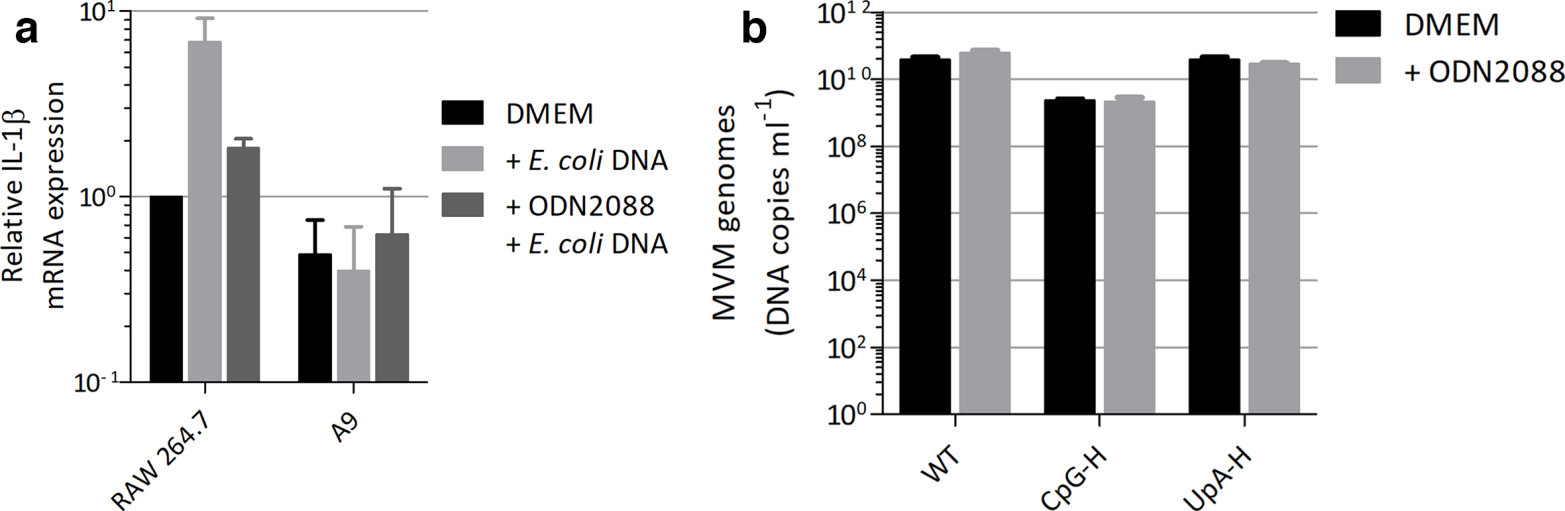
Involvement of TLR9 in MVM mutant phenotype. (a) IL-1β induction after TLR9 induction by purified *E. coli* DNA in the presence or absence of TLR-9 antagonist ODN2088 . *n*=3, +/−sem. b) Viral genome copies 72 h post-infection with 1000 viral genome copies/cell in the presence or absence of ODN2088. *n*=3.

It has been previously proposed that non-methylated CpG sites in parvovirus genomic DNA activate a cellular antiviral response through Toll-like receptor 9 (TLR9) and it has been shown that MVM can be recognized by TLR9 in human PBMCs and HEK-hTLR9 cells, but not murine bone marrow cells or plasmacytoid dendritic cells [43, 44]. To investigate whether TLR9 mediated the attenuated phenotype of CpG-high mutants of MVM, we first determined whether the A9 cells in which CpG-mediated attenuation was most evident can actually signal through TLR9. IL-1β mRNA levels were measured before and after stimulation with *E. coli* DNA, a TLR-9-specific stimulant (Fig. 9a). In the same experiment, we also tested the efficacy of the TLR-9-specific antagonist ODN2088. This is a 15-mer oligodinucleotide (5′-TCC TGG CGG GGA AGT-3′) that inhibits binding of unmethylated CpG sequences to TLR9 [45].

As a positive control, RAW264.7 cells were responsive to TLR9 stimulation, with 2 h pre-treatment with ODN2088 inhibiting most of this response. In contrast, A9 cells showed no induction of IL-1 β mRNA. We additionally assayed the potential effects of TLR9 expression on attenuation in a functional assay. Replication of WT and the CpG-H first half-mutant was compared between A9 cells and those pretreated with ODN2088. Two hours’ pretreatment of A9 cells with ODN2088 had no or minimal effects on the replication of WT or TpA-H MVM (Fig. 9b). Significantly, there was no relevant recovery of replication ability of the CpG-H first half-mutant, thereby providing no evidence that TLR9 controls its replication in the A9 cell line.

ZAP has previously been shown to participate in the attenuation of mutants of HIV-1 with elevated CpG frequencies. Similarities in the replication strategy of parvoviruses and retroviruses suggest a potential role for ZAP in mediating the restriction of CpG-modified mutants of MVM. We have previously constructed ZAP k/o A549 cell lines to investigate the attenuation pathways of E7 [21]. Replication of MVM in this human cell line was less efficient than in A9 cells, although both mRNA and protein expression and limited genomic replication occurred and could be quantified.

**Fig. 10.**
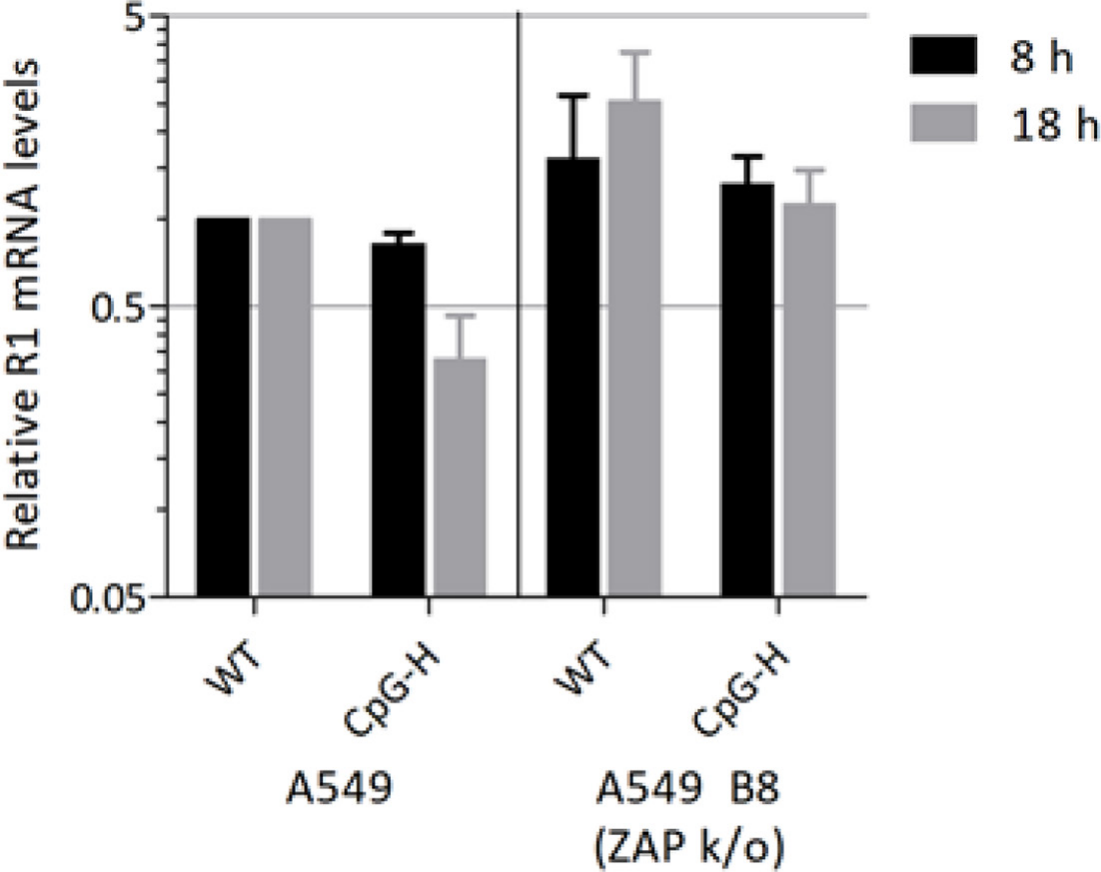
MVM R1 mRNA levels in A549 andA549 B8 (ZAP k/o) cell lines post-transfection. MVM R1 mRNA expression 8 and 18 h post-transfection normalized to cellular GAPDH and MVM WT R1 levels in A549 cells. PlasmidpMM984, viral infectious clone, replication inhibited by Aphidicolin. *n*=3, +/−sem.

To quantify the effects of ZAP expression on MVM RNA levels, A549 and B8 (ZAP k/o) cells were transfected with DNA sequences of WT and CpG-H mutants of MVM in the presence of aphidicolin; R1 mRNA transcript levels were then quantified by qPCR at 8 and 18 h (Fig. 10). As observed previously in A9 cells (Fig. 7), mRNA levels were comparable in A549s at 8 h, but then declined by approximately fourfold at 18 h. In contrast, there was no decline in RNA levels of the CpG-H mutant between 8 and 18 h in transfected B8 cells, indicating a potential role for ZAP in the observed reduced expression or instability of CpG-high RNA sequences in this context.

**Fig. 11.**
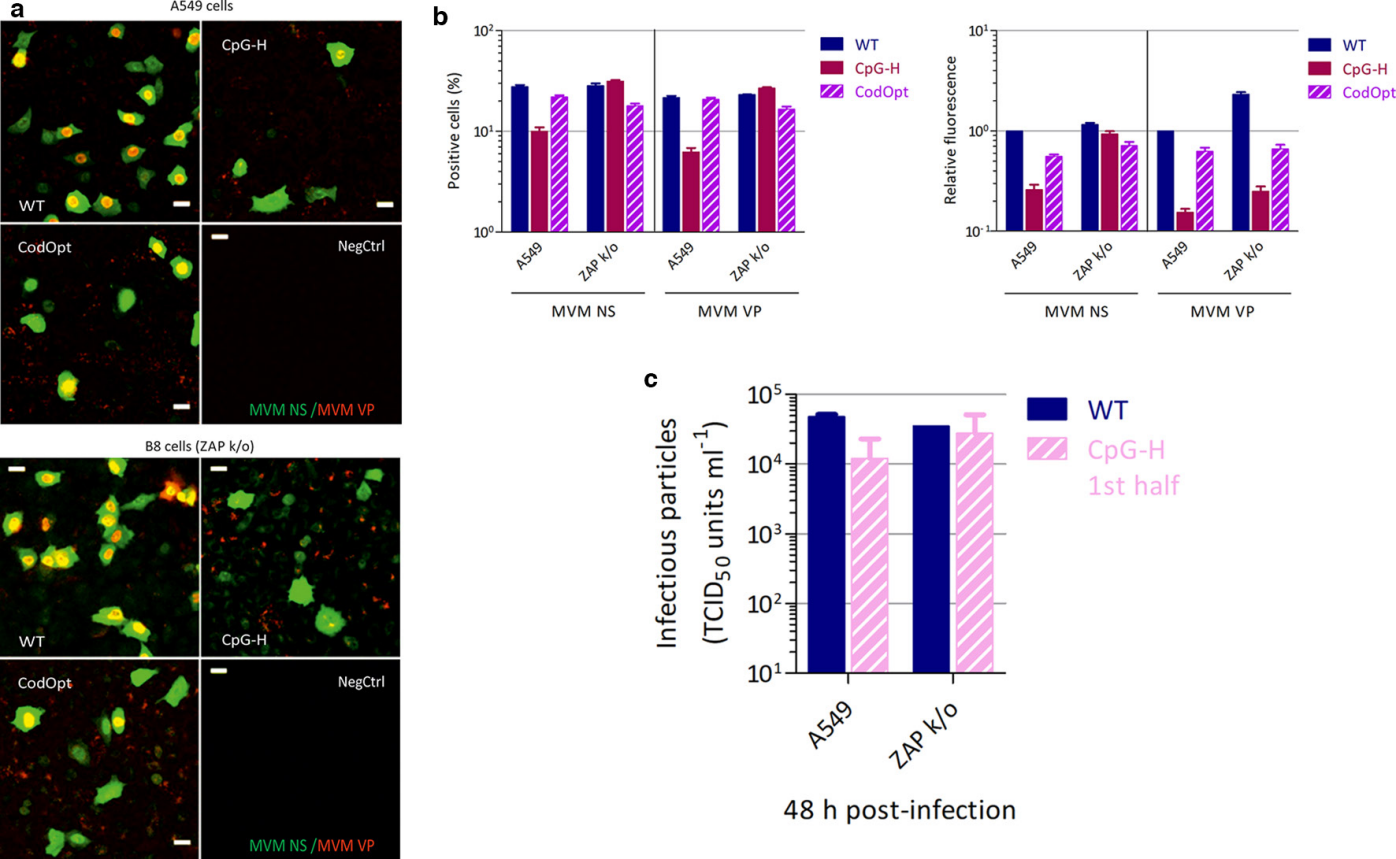
Cellular distribution of MVM viral proteins in A549 and B8 (ZAP k/o) cells . (a) Immunofluorescence staining of viral NS (green) and VP (red) proteins at 18 h post-infection with 1000 viral copies/cell. Uninfected cells were used as a negative control. (b) Quantification of immunofluorescence staining of viral NS and VP proteins using Imaris and Fiji software separately for the frequency of cells expressing NS or VP (left) or relative fluorescence intensity of NS or VP compared to WT MVM (right). Bar heights represent the mean of three biological replicates; error bars show sems. (c) Replication of WT and CpG-H first half-mutant of MVM in A549 and ZAP k/o cells infected at an m.o.i. of 1. Cell supernatant was harvested at 48 h and infectivity was determined by titration on NB324K cells. Bar heights show the mean of two replicates; error bars show the sd.

Further evidence for a role of ZAP in CpG-mediated attenuation was provided by quantitation of the virus replication of MVM and CpG-high mutants by immunocytochemistry and replication kinetics in the B8 ZAP k/o cell line. A549 and B8 cells were infected with WT, CpG-H or CodOpt virus and stained for both NS and VP proteins (representative sections are shown in Fig. 11a, full panel and close up in Figs S6 and S7). The frequencies and fluorescent intensities of stained capsid and NS proteins were separately quantified (Fig. 11b). The frequency of cells expressing NS or VP was reduced threefold for the CpG-H mutant in A549 cells but with a much lesser reduction in infection frequencies of the codon-optimized mutant. Similarly, the average intensity of cells infected with the CpG-H mutant was reduced 4-fold for the NS proteins and 9-fold for the VP proteins, while CodOpt was reduced 1.5–2-fold. In contrast, the proportion of B8 ZAP k/o cells expressing NS or VP was comparable to that of the WT virus, effectively reversing the restricted phenotype observed in A549 cells. The average fluorescence intensity of NS in B8 cells infected with the CpG-H mutants was also restored to WT levels (Fig. 11b). There was also restoration of VP expression, but remarkably there was no relocalization of VP to the nucleus of ZAP k/o cells, with protein remaining in punctate granules in the cytoplasm. These findings indicate that ZAP plays a role in restricting VP expression of CpG-high virus mutants, but other mechanisms also influenced by RNA composition potentially play roles in subsequent steps in MVM replication.

Infection of A549 cells led to a full infection cycle of MVM and produced detectable levels of infectious particles at 48 h post-infection, albeit at a lower level than in other cell lines (Figs. 8c, 11a). Replication of the CpG-H first half-mutant was impaired compared to WT in the A549 cell line, but not in the B8 ZAP k/o cell line (Fig. 11c).

## Discussion

This study was motivated by the observation that the suppression of CpG dinucleotide frequencies found in vertebrate RNA viruses and HIV-1 also occurs in small DNA viruses, such as parvoviruses, polyomaviruses and papillomaviruses [10, 12, 46]. In the current study, we document a remarkable attenuation of the replication ability of MVM constructs with artificially increased frequencies of CpG dinucleotides in the VP2 gene, with several log reductions in replication kinetics compared to WT virus and a detectable frequency of reversion of CpG sites on passaging CpG-H mutant viruses. The study used a variety of approaches to investigate the mechanism for this attenuation in the context of previous descriptions of potential effects of CpG addition on genome methylation, translation efficiency and mRNA stability.

It has previously been hypothesized that the suppression of CpG frequencies in parvoviruses and potentially other small DNA viruses results from methylation-induced hypermutation by cellular methyltransferases in the nuclear compartment. Such processes may reduce the visibility of DNA viruses to TLR9, which acts as a bacterial and viral pattern recognition receptor through its specific recognition of non-methylated CpG dinucleotides in DNA sequences. However, it has been since shown that the genome of porcine parvovirus (PPV) is hypo-methylated throughout its replication cycle, both in replicating DNA complexes and genomic DNA packaged into virions; genomes of adenoviruses, papillomaviruses and polyomaviruses are similarly methylation deficient [47–51]. Artificial methylation of PPV genomes prior to transfection showed a small inhibitory rather than enhancing effect on replication initiation and their infectious progeny became non-methylated.

MVM and parvovirus H-1 could be detected by TLR9 in human PBMCs and HEK-hTLR9 MVM, but adenovirus association virus 2 remained unrecognized by TLR9 expressed at high level on murine plasmacytoid dendritic cells [43, 44]. A further demonstration of a lack of involvement of TLR9 in CpG suppression was provided by our own observations that the A9 cell line in which CpG-H mutants of MVM were attenuated showed no induction of IL-1β after TLR9 stimulation and no reversal of attenuation when TLR9 signalling was inhibited with an TLR9 antagonist (Fig. 9). As previously proposed, it appears that the suppression of CpG dinucleotides in parvovirus genomes originates from a different selection or mutational pressure from that which drives vertebrate genomic CpG under-representation, although further attenuation through TLR9 involvement cannot be excluded in cell lines with a functional TLR9 pathway.

In the current study we have investigated other potential mechanisms underlying the observed suppression of CpG dinucleotide frequencies in MVM and other parvoviruses. In a range of RNA viruses, it has previously been proposed that CpG suppression reflects a process of avoiding unfavoured codons and codon pairs that contain CpG dinucleotides within codons or across codon boundaries [52–55]. Their presence may reduce translation rates from the mRNA template and restrict virus replication. However, the observation of high levels of attenuation of a mutant of MVM with a codon-optimized capsid gene is entirely inconsistent with the proposed translational basis for attenuation. While the CodOpt RNA template sequence translated more efficiently in a cell-free assay to the native WT sequence (Fig. 6b, c), the mutant virus constructed with this capsid sequence showed extreme attenuation (Figs. 4 and 6a), more compatible with effects of the concomitant increase in the number of CpG dinucleotides (WT: 7, CodOpt: 70; Table 1) and an increase in G+C content, which additionally influenced replication (Fig. 3). These findings are consistent with our previous analysis of mutants of E7 in which CpG and codon pair bias were independently manipulated with the outcome that attenuation originated from CpG and UpA frequency changes and the effects of codon pair changes were secondary to that [17]. They also concur with our previous observations that CpG (and UpA) dinucleotides added to a non-coding region of an E7 replicon construct produced similar attenuation to mutants where the same compositional modification was made to the coding region [56].

CpG-mediated attenuation of MVM was dose-dependent, with the half-mutants with only 58 or 59 CpGs added showing around 1000-fold enhanced replication compared to the full CpG-H mutant (Fig. 3a, b), although remaining 1000-fold less replication-competent than WT virus. This dose dependence potentially accounts for the previous failure to observe any substantial attenuation of parvovirus H-1 and porcine parvovirus with far fewer CpGs introduced into their genome (5–13 and 9–29, respectively). A contributory factor was the use of kidney-derived cell lines (NB324K and HEK293) that, along with BHK, show far less CpG-associated attenuation than fibroblast cell cultures [21, 56]. Construction of further mutants with a wider range of CpG compositions would be required to determine whether there was a minimum threshold concentration/spacing to achieve the CpG-mediated attenuation observed in the current study. Despite the severe attenuation, CpG-H mutants remained remarkably stable during *in vitro* passage (Fig. S3), indicating that the virus cannot readily escape the detrimental mutations while remaining viable.

STING has been identified as member of the principal pathway activated by a range of cytoplasmic pattern recognition receptors (PRRs) for viral DNA (reviewed in [57, 58]). Although undescribed to date, it is possible that there exist PRRs for viral genomic DNA with a sequence specificity for CpG that signal through STING. To investigate this, we investigated the susceptibility of STING and a range of other pathway k/os in MEF cells to MVM and a range of compositionally modified mutants. Parental MEFs did not support MVM replication, but MEFs with knockouts of PKR, STING and interferon α receptor (IFNAR) supported a high level replication, indicative of their potent roles in restricting parvovirus replication (Fig. 8a). The observed susceptibility of PKR k/o cells to MVM is consistent with previous observations [59]. While these three pathways substantially restricted MVM replication, there was no evidence for any dinucleotide compositional specificity in their restriction, with k/o cells showing equivalent attenuation of CpG-H mutant replication to that observed in A9 cells. Given the centrality of these three proteins and associated pathways in the innate cellular response to virus infection, these observations effectively rule out a vast swathe of DNA PRRs and other activators of IRF3- or IRF7-induced interferon activated pathways in CpG-mediated restriction, including TLR9 (Fig. 8b).

If not through cytoplasmic PRRs for genomic DNA, CpG suppression in parvoviruses may alternatively originate from pathways recognizing CpG dinucleotides in viral mRNA sequences expressed during the replication cycle [18, 19, 56, 60]. Supporting this possibility are the data we obtained that document a role of ZAP in mediating the restriction of CpG-high mutants of MVM (Figs. 10 and 11), a pathway shown previously to be responsible for the attenuation of CpG-high mutants of HIV-1 [19] and in a different RNA virus model based on E7 [21]. Concerning the former, there are indeed several parallels in the strategy for gene expression of HIV-1 and parvoviruses, in nuclear RNA transcription and export of mRNAs into the cytoplasm, and therefore potentially in the steps in their replication targeted by ZAP. For HIV-1, ZAP has been shown to specifically target and bind to CpG sites in mRNA sequences of CpG-high mutants [19], needing KHNYN protein as a co-factor to exert its antiviral activity against CpG-rich RNAs [20]. The observation of higher rates of degradation of CpG-high MVM sequences in A549 cells (Fig. 10) and its reversion of ZAP k/o cells supports the involvement of this pathway in the attenuation of MVM. This is further indicated by restoration of NS and partial restoration of capsid gene expression in B8 cells from CpG-high MVM mutants on immunocytochemistry and of replication in A549 cells (Fig. 11).

However, a difference between the data collected for MVM in the current study and previous analyses of E7 and influenza A virus was the consistent absence of attenuation of the TpA-high mutant of MVM (Figs. 3–7). The increase in TpA dinucleotides achieved by mutagenesis and the proportion of the genome modified (24.4 %) were comparable to those for E7, in which the UpA-high mutant showed an approximately 100-fold reduction in replication kinetics in an assay format comparable to that used for MVM (Fig. 3). The replication of the TpA-high mutant of MVM was similar to that for the WT in all other assay formats (Figs. 4–7), and did not show any evidence for the reduction in RNA stability observed for CpG-enriched sequences (Fig. 7). It is possible that this reflects the outcome of different restriction mechanisms responding to high CpG and TpA sequences and the inactivity of the latter pathways in parvovirus replication. However, our previous analyses provided consistent evidence that the pathway restricting CpG- and UpA-high mutants is shared. In recently completed work [21], we indeed demonstrated that ZAP is responsible for the attenuated phenotype of both CpG- and UpA-high mutants; ZAP furthermore shows a similar binding affinity to E7 viral RNA transcripts with inserted UpA-high or CpG-high regions. How the corresponding TpA-high mutant of MVM escapes these mechanisms operating at the RNA level on viral mRNAs is therefore currently unclear.

The marked attenuation of the codon-optimized mutant of MVM (Fig. 3) was unexpected and remarkable. Codon optimization is widely used to enhance the expression of transgenes in a variety of DNA and virus vectors, including other members of the *Parvoviridae*, such as adeno-associated viruses (AAVs) [61–63]. However, it appears that in MVM and perhaps in other small DNA viruses and retroviruses with suppressed CpG frequencies, codon optimization has a severely damaging effect on expression (Fig. 4). A similar effect has been observed in adenovirus, where most viral proteins are codon-optimized, but optimizing the non-optimized fibre protein led to a decrease in viral fitness [64].

In summary, this study provides compelling evidence for the action of cellular restriction of CpG-modified mutants of MVM operating at the RNA level, attenuating and affecting all downstream stages of the viral replication cycle. The study provides further evidence for a commonality in restriction mechanisms and their effect on driving down CpG frequencies in most RNA viruses and small DNA viruses infecting vertebrates, rather than through previously proposed TLR9 recognition.

## Supplementary Data

Supplementary material 1
