# Supplementary material for: Use of a small DNA virus model to investigate mechanisms of CpG dinucleotide-induced attenuation of virus replication
Source: J Gen Virol. 2020 Aug 12;101(11):1202–18. doi: 10.1099/jgv.0.001477 (PMC7879557; doi:10.1099/jgv.0.001477)
Supplement: Supplementary material 1 [file jgv-101-1202-s001.pdf]

SUPPLEMENTARY DATA

TABLE S1

Sequences of VP inserts and expression plasmids used in the study

A. Inserts

>CLDR

ATGGCCAAAGATGATGCACATGAGCAAATTTGGACACCATGGAGCTTGGTTGATGCAAATGCGTGGGGAGTTTGGCTTCAACCAAGTGACTGGCAATAC
ATTTGCAACACAATGAGTCAACTTAATTTGGTTTCACTTGATCAAGAAATATTCAATGTAGTGTTGAAAACTGTGACAGAGCAAGACTTAGGAGGACAAGC
TATAAAAATATACAACAATGACCTCACAGCATGTATGATGGTAGCAGTAGATTCTAACAACATATTGCCATACACTCCAGCAGCAAACTCAATGGAAACTC
TTGGATTCTACCCATGGAAACCTACTATTGCTTCCCCATACAGATACTATTTTTGCGTTGACAGAGACCTATCAGTGACATATGAAAATCAAGAAGGCACTG
TTGAACATAATGTCATGGGAACACCAAAAGGAATGAACTCACAATTTTTTACCATTGAGAACACACAGCAAATCACCTTGCTGAGAACTGGGGACGAATTT
GCCACAGGCACCTACTACTTTGACACAAATTCAGTTAAACTTACACACACTTGGCAAACAAACCGTCAACTAGGACAGCCTCCACTTCTTTCAACATTTCCT
GAAGCAGACACTGATGCAGGTACACTAACTGCACAAGGAAGCAGACATGGGACAACTCAAATGGGAGTTAACTGGGTTAGTGAAGCCATAAGAACCAG
ACCAGCTCAAGTAGGCTTTTGTCAGCCACACAATGACTTTGAAGCAAGCAGGGCAGGCCCATTTGCAGCACCTAAAGTTCCAGCTGACATTACACAAGGA
GTGGACAAAGAAGCCAATGGTAGTGTTAGATACAGCTATGGCAAGCAGCATGGTGAAAATTGGGCTTCACATGGACCTGCACCTGAACGTTACACATGG
GATGAAACAAGTTTTGGTTCTGGCAGAGACACAAAAGACGGCTTCATTCAATCTGCACCACTAGTTGTGCCACCACCTCTAAATGGCATCCTTACCAATGC
AAATCCAATTGGGACAAAAAATGACATTCACTTTTCAAATGTTTTTAACAGTTATGGACCTCTAACTGCTTTTTCACACCCAAGTCCAGTGTACCCTCAAGGT
CAAATATGGGATAAAGAACTAGATCTTGAACACAAACCCAGACTTCACATTACTGCACCCTTTGTTTGTAAAAACAATGCACCTGGACAAATGTTGGTTAG
GCTAGGACCCAACCTTACAGACCAATATGATCCCAACGGAGCAACACTTT

>GpC-H

ATGGCAAAAGATGATGCTCATGAGCAAATTTGGACACCATGGAGCTTGGTTGATGCTAATGCTTGGGGAGTTTGGCTGCAGCCAAGTGACTGGCAATACA
TTTGCAACACAATGAGCCAGCTTAACTTGGTATCACTTGATCAAGAAATATTCAATGTAGTGCTCAAAACTGTTACAGAACAAGACTTAGGAGGGCAAGCT
ATAAAAATATACAACAATGACCTTACAGCTTGCATGATGGTTGCAGTAGATTCAAACAACATTTTGCCATACACTCCTGCAGCAAACTCAATGGAAACACTT
GGATTTTACCCATGGAAGCCAACAATAGCATCACCATACAGGTATTATTTTTGCGTTGACAGAGATCTTTCAGTCACATACGAAAATCAAGAAGGCACAGT
TGAGCATAATGTCATGGGTACACCAAAAGGCATGAATTCTCAATTTTTTACAATTGAAAACACACAGCAAATCACATTGCTGAGAACAGGTGATGAATTTG
CAACAGGCACTTACTACTTTGACACAAATTCAGTTAAGCTTACACACACTTGGCAAACAAACCGCCAGCTTGGCCAGCCTCCACTGCTTTCAACATTTCCTG
AAGCTGACACTGATGCAGGAACACTTACTGCACAAGGCAGCAGGCATGGCACAACACAAATGGGTGTTAACTGGGTTAGCGAAGCAATTAGAACAAGGC
CTGCTCAAGTAGGCTTTTGCCAGCCACACAATGATTTTGAAGCAAGCAGAGCTGGCCCATTTGCTGCTCCAAAAGTGCCAGCAGATATTACACAAGGAGTA
GACAAAGAAGCAAATGGCAGTGTTAGATACAGCTATGGCAAGCAGCATGGTGAAAATTGGGCTTCACATGGCCCAGCACCAGAGCGATACACATGGGAT
GAAACAAGCTTTGGGTCAGGCAGAGACACAAAAGATGGTTTTATTCAATCAGCACCACTAGTTGTGCCACCACCACTAAATGGAATTCTTACAAATGCAAA
CCCTATTGGAACTAAAAATGACATTCATTTTTCAAATGTTTTTAACAGCTATGGCCCACTAACTGCATTTTCACACCCAAGCCCTGTATATCCTCAAGGGCAA
ATATGGGACAAAGAACTAGATCTTGAACATAAGCCTAGGCTGCACATAACTGCGCCATTTGTTTGCAAAAACAATGCACCTGGCCAAATGTTGGTTAGATT
AGGCCCAAATCTAACTGACCAATATGATCCAAACGGAGCAACACTTT

>TpA-H

ATGGCTAAAGATGATGCACATGAGCAAATATGGACACCATGGAGCTTAGTAGATGCTAATGCTTGGGGAGTATGGCTACAGCCCAGTGACTGGCAATAC
ATATGCAACACCATGAGCCAGCTTAACTTAGTATCACTAGATCAAGAAATATTCAATGTAGTCCTTAAAACAGTTACAGAGCAAGACTTAGGAGGACAAGC
TATAAAGATATACAATAATGACCTTACAGCCTGCATGATGGTAGCAGTAGACTCTAACAACATATTACCATATACACCTGCAGCTAACTCTATGGAAACCCT
AGGTTTCTACCCCTGGAAGCCTACTATAGCATCACCATACAGGTACTATTTTTGCGTGGACAGAGATCTATCAGTTACCTACGAGAATCAAGAGGGCACAG
TAGAACATAATGTTATGGGCACCCCTAAAGGTATGAATTCTCAATTTTTTACTATAGAGAATACACAACAAATTACATTACTTAGAACAGGGGACGAATTT
GCCACAGGTACATACTACTTTGATACAAATTCAGTTAAACTTACACATACGTGGCAAACCAACCGTCAGCTAGGACAGCCTCCACTACTATCTACCTTTCCT
GAAGCTGATACTGATGCAGGTACACTTACTGCCCAAGGTAGCAGGCATGGTACTACACAAATGGGGGTTAACTGGGTTAGTGAAGCTATTAGAACCAGA
CCAGCCCAAGTAGGATTTTGTCAACCACACAATGACTTTGAAGCTAGCAGAGCTGGACCATTTGCTGCCCCCAAAGTACCAGCAGATATTACTCAAGGAGT

AGACAAAGAAGCCAATGGTAGTGTCAGGTACAGTTATGGCAAACAGCATGGGGAAAATTGGGCTTCCCATGGACCAGCACCAGAGCGCTACACCTGGGA
TGAAACTAGCTTTGGTTCAGGTAGAGACACTAAGGATGGTTTTATACAATCAGCCCCACTAGTGGTACCACCACCACTAAATGGCATACTCACTAATGCAA
ACCCTATAGGTACTAAAAATGACATACATTTTTCCAATGTATTTAATAGCTATGGGCCCCTAACAGCATTTTCACACCCCAGTCCAGTCTACCCTCAGGGAC
AAATATGGGACAAAGAACTAGATCTTGAACATAAACCTAGACTACACATAACTGCTCCATTTGTATGTAAAAATAATGCCCCAGGGCAAATGTTAGTTAGA
TTAGGACCTAACCTAACTGACCAATATGATCCTAACGGAGCCACACTGT

CpG-H

>*CpG-H1*

ATGGCAAAAGACGACGCACACGAGCAAATTTGGACACCGTGGAGCTTGGTTGACGCGAACGCGTGGGGTGTTTGGCTACAGCCAAGCGACTGGCAATAC
ATTTGCAACACTATGAGCCAGCTTAACTTGGTATCGCTTGATCAAGAAATATTCAATGTTGTACTAAAAACCGTTACCGAGCAAGACTTAGGTGGTCAAGC
AATAAAAATATACAACAATGACCTTACCGCGTGCATGATGGTTGCCGTTGACTCAAACAACATTTTGCCGTACACGCCCGCCGCGAACTCGATGGAAACTC
TTGGTTTTTACCCGTGGAAACCGACAATAGCGTCGCCATACCGTTACTATTTTTGCGTTGACCGTGATCTTTCCGTTACGTACGAAAATCAAGAAGGAACCG
TTGAACATAACGTAATGGGAACGCCAAAAGGAATGAATTCACAATTTTTTACGATAGAGAACACGCAACAAATAACGTTGCTACGAACCGGTGACGAATT
TGCAACTGGTACGTACTACTTCGACACGAATTCGGTTAAACTAACGCACACGTGGCAAACAAACCGTCAACTTGGACAACCGCCGCTTCTTTCAACATTTCC
CGAAGCCGACACGGACGCGGGTACACTTACCGCACAAGGTAGCCGACACGGAACGACGCAAATGGGAGTT

>*CpG-H2*

AACTGGGTAAGTGAAGCAATACGAACACGACCCGCACAAGTCGGATTTTGTCAACCACACAACGACTTTGAAGCAAGCCGTGCCGGACCGTTTGCAGCGC
CGAAAGTTCCTGCCGATATTACGCAAGGTGTTGACAAAGAAGCAAATGGAAGTGTTCGATACAGTTACGGAAAACAACATGGTGAAAATTGGGCGTCGC
ACGGACCGGCGCCCGAACGATACACGTGGGATGAAACGAGCTTCGGTTCGGGTCGTGACACGAAAGATGGTTTTATTCAATCCGCGCCGCTCGTTGTTCC
ACCGCCGCTAAACGGAATTCTTACGAACGCGAACCCGATTGGAACCAAAAACGACATTCATTTTTCTAACGTTTTTAACAGCTATGGTCCACTAACCGCGTT
TTCGCACCCGAGTCCCGTATACCCACAAGGACAAATATGGGACAAAGAACTTGATCTTGAACACAAACCGCGACTTCACATAACTGCGCCATTCGTTTGTA
AAAACAACGCGCCCGGACAAATGTTGGTTCGATTAGGACCAAACCTAACCGACCAATACGATCCGAACGGTGCAACGCTTT

>CpG-H 42% G+C

ATGGCAAAAGACGACGCACACGAACAAATTTGGACACCGTGGAGTTTGGTTGACGCAAACGCATGGGGCGTTTGGCTACAACCAAGCGATTGGCAATAC
ATTTGCAACACAATGAGCCAACTTAATTTGGTATCGCTAGATCAAGAAATATTCAACGTTGTTCTTAAAACTGTTACAGAACAAGATTTAGGCGGTCAAGC
GATAAAAATATACAACAATGATCTTACAGCATGCATGATGGTTGCAGTTGATTCAAACAACATTTTGCCTTACACACCAGCTGCAAATTCAATGGAAACACT
TGGTTTTTATCCATGGAAACCAACGATAGCATCGCCGTATCGTTATTATTTTTGCGTTGATCGCGATCTTTCTGTAACGTACGAAAATCAAGAAGGAACAGT
TGAACATAATGTAATGGGAACACCAAAAGGAATGAATTCGCAATTTTTTACTATAGAAAACACGCAACAAATAACATTGCTACGAACGGGCGACGAATTC
GCGACAGGTACATATTATTTTGACACAAATTCCGTTAAACTAACTCACACATGGCAAACAAATCGTCAACTTGGACAACCGCCACTTCTATCAACATTTCCC
GAAGCGGACACAGACGCAGGTACACTTACCGCGCAAGGTAGTCGACATGGAACAACACAAATGGGCGTTAACTGGGTTAGCGAAGCAATACGAACACG
ACCTGCACAAGTTGGATTTTGTCAACCACATAACGATTTCGAAGCAAGTCGCGCTGGACCATTCGCGGCGCCGAAAGTTCCTGCCGATATTACACAAGGA
GTTGACAAAGAAGCAAACGGAAGCGTTCGATACAGTTACGGAAAACAACACGGAGAAAATTGGGCATCGCATGGACCGGCACCAGAACGATACACATG
GGACGAAACGAGTTTCGGTTCCGGTCGCGACACAAAAGACGGTTTTATTCAATCTGCACCACTTGTTGTTCCTCCACCTCTAAATGGAATTCTTACAAATGC
AAATCCAATCGGAACAAAAAACGACATTCATTTTTCAAACGTTTTTAACAGTTATGGTCCTCTAACAGCGTTTTCTCATCCGAGTCCTGTATATCCGCAAGG
ACAAATATGGGACAAAGAACTTGATCTTGAACACAAACCACGACTTCACATAACTGCTCCTTTCGTTTGTAAAAACAACGCACCTGGACAAATGTTGGTTC
GATTAGGACCAAATCTAACTGATCAATACGATCCAAACGGAGCAACTCTTT

>CodOpt

ATGGCCAAGGACGACGCCCACGAGCAGATCTGGACACCTTGGAGCCTGGTGGACGCCAACGCTTGGGGAGTGTGGCTGCAGCCTAGCGACTGGCAGTA
CATCTGCAACACCATGAGCCAGCTGAATCTGGTGTCCCTGGACCAGGAAATCTTCAACGTGGTGCTGAAAACCGTGACCGAGCAGGACCTGGGCGGCCA
GGCCATCAAGATCTACAACAACGACCTGACCGCCTGCATGATGGTGGCCGTGGACAGCAACAACATCCTGCCCTACACCCCTGCCGCCAACAGCATGGAA
ACCCTGGGCTTCTACCCCTGGAAGCCCACAATCGCCAGCCCCTACCGGTACTACTTCTGCGTGGACAGGGACCTGTCCGTGACCTACGAGAACCAGGAAG
GCACCGTGGAACACAACGTGATGGGCACCCCCAAGGGCATGAACAGCCAGTTCTTCACCATCGAGAACACCCAGCAGATCACCCTGCTGAGAACCGGCG
ACGAGTTCGCCACCGGCACCTACTACTTCGACACCAACAGCGTGAAGCTGACCCACACCTGGCAGACCAACAGACAGCTGGGCCAGCCTCCTCTGCTGAG
CACATTCCCTGAGGCCGATACCGACGCCGGCACACTGACAGCACAGGGCAGCAGACACGGCACCACCCAGATGGGCGTTAACTGGGTGTCCGAGGCCAT
CAGAACCAGACCTGCCCAAGTGGGCTTCTGCCAGCCCCACAACGATTTCGAGGCCAGCAGAGCCGGCCCTTTCGCCGCTCCTAAAGTGCCTGCCGACATC
ACCCAGGGCGTGGACAAAGAGGCTAACGGCAGCGTGCGGTACAGCTACGGAAAGCAGCACGGCGAGAACTGGGCCTCTCACGGACCTGCCCCTGAGAG
ATACACCTGGGACGAGACAAGCTTCGGCAGCGGCAGAGACACCAAGGACGGCTTCATCCAGAGCGCCCCACTGGTGGTGCCTCCACCTCTGAACGGCAT
CCTGACCAACGCCAACCCCATCGGCACCAAGAACGACATCCACTTCAGCAACGTGTTCAACTCCTACGGCCCCCTGACAGCCTTCAGCCACCCCAGCCCAG
TGTATCCACAGGGACAGATCTGGGACAAAGAACTGGACCTGGAACATAAGCCCAGACTGCACATCACCGCCCCCTTCGTGTGCAAGAACAACGCCCCTGG
CCAGATGCTCGTGCGGCTGGGACCTAACCTGACCGACCAGTACGATCCCAACGGCGCCACCCTGT

>CodOpt CpG-L

ATGGCCAAGGATGATGCCCACGAGCAGATCTGGACCCCCTGGAGCCTGGTGGATGCCAATGCCTGGGGGGTGTGGCTGCAGCCCTCTGACTGGCAGTAC
ATCTGCAATACCATGAGCCAGCTGAATCTGGTGTCCCTGGATCAGGAGATCTTCAATGTGGTGCTGAAGACTGTGACAGAGCAGGACCTGGGAGGCCAG
GCCATCAAGATCTACAACAATGACCTGACAGCCTGTATGATGGTGGCCGTGGACTCCAACAACATCCTGCCTTACACACCAGCTGCCAACAGCATGGAGA
CCCTGGGCTTCTACCCATGGAAGCCCACCATTGCCTCCCCATACAGGTACTACTTCTGTGTGGACAGAGACCTCTCAGTGACCTATGAGAACCAGGAGGGC
ACAGTGGAGCACAATGTGATGGGAACCCCAAAGGGCATGAATAGCCAGTTTTTCACCATCGAGAATACCCAGCAGATCACCCTGCTGAGAACCGGGGAT
GAGTTTGCCACAGGCACCTACTACTTTGACACCAACTCTGTGAAGCTGACCCACACATGGCAGACCAACAGACAGCTGGGCCAGCCCCCCCTGCTGTCCAC
CTTCCCTGAGGCTGACACAGATGCCGGCACCCTGACAGCCCAGGGCTCCAGACATGGCACCACCCAGATGGGAGTTAACTGGGTGTCAGAGGCCATCAG
AACCAGGCCTGCCCAGGTGGGATTCTGCCAGCCCCACAACGACTTTGAGGCCTCCAGGGCAGGCCCCTTTGCTGCCCCTAAAGTGCCAGCTGACATCACC
CAGGGAGTGGACAAGGAGGCCAATGGCTCTGTGAGATACTCATATGGCAAGCAGCATGGCGAGAACTGGGCCAGCCATGGACCTGCCCCAGAGAGATA
CACTTGGGATGAGACCAGCTTTGGCTCTGGCAGAGACACCAAAGATGGCTTCATCCAGTCTGCCCCCCTGGTGGTGCCCCCTCCACTGAATGGAATCCTGA
CCAATGCCAATCCAATTGGCACCAAGAATGACATTCACTTCAGCAATGTGTTCAACAGCTATGGCCCCCTGACAGCCTTTAGCCACCCCTCCCCTGTGTATC
CACAGGGGCAGATCTGGGACAAGGAGCTGGACCTGGAGCACAAGCCAAGACTGCACATCACAGCCCCATTTGTGTGCAAGAATAATGCCCCTGGCCAGA
TGCTGGTGAGACTGGGCCCTAACCTGACAGACCAGTATGACCCCAATGGGGCCACCCTGT

B. pCAG_MVMp expression plasmid

Insert region highlighted in grey

>pCAG_MVM_R3

GATTCGACATTGATTATTGACTAGTTATTAATAGTAATCAATTACGGGGTCATTAGTTCATAGCCCATATATGGAGTTCCGCGTTACATAACTTACGGTAAA
TGGCCCGCCTGGCTGACCGCCCAACGACCCCCGCCCATTGACGTCAATAATGACGTATGTTCCCATAGTAACGCCAATAGGGACTTTCCATTGACGTCAAT
GGGTGGAGTATTTACGGTAAACTGCCCACTTGGCAGTACATCAAGTGTATCATATGCCAAGTACGCCCCCTATTGACGTCAATGACGGTAAATGGCCCGCC
TGGCATTATGCCCAGTACATGACCTTATGGGACTTTCCTACTTGGCAGTACATCTACGTATTAGTCATCGCTATTACCATGGTCGAGGTGAGCCCCACGTTC
TGCTTCACTCTCCCCATCTCCCCCCCCTCCCCACCCCCAATTTTGTATTTATTTATTTTTTAATTATTTTGTGCAGCGATGGGGGCGGGGGGGGGGGGGGG
CGCGCGCCAGGCGGGGCGGGGCGGGGCGAGGGGCGGGGCGGGGCGAGGCGGAGAGGTGCGGCGGCAGCCAATCAGAGCGGCGCGCTCCGAAAGTT
TCCTTTTATGGCGAGGCGGCGGCGGCGGCGGCCCTATAAAAAGCGAAGCGCGCGGCGGGCGGGAGTCGCTGCGTTGCCTTCGCCCCGTGCCCCGCTCCG
CGCCGCCTCGCGCCGCCCGCCCCGGCTCTGACTGACCGCGTTACTCCCACAGGTGAGCGGGCGGGACGGCCCTTCTCCTCCGGGCTGTAATTAGCGCTTG
GTTTAATGACGGCTCGTTTCTTTTCTGTGGCTGCGTGAAAGCCTTAAAGGGCTCCGGGAGGGCCCTTTGTGCGGGGGGGAGCGGCTCGGGGGGTGCGTG
CGTGTGTGTGTGCGTGGGGAGCGCCGCGTGCGGCCCGCGCTGCCCGGCGGCTGTGAGCGCTGCGGGCGCGGCGCGGGGCTTTGTGCGCTCCGCGTGTG
CGCGAGGGGAGCGCGGCCGGGGGCGGTGCCCCGCGGTGCGGGGGGGCTGCGAGGGGAACAAAGGCTGCGTGCGGGGTGTGTGCGTGGGGGGGTGA
GCAGGGGGTGTGGGCGCGGCGGTCGGGCTGTAACCCCCCCCTGCACCCCCCTCCCCGAGTTGCTGAGCACGGCCCGGCTTCGGGTGCGGGGCTCCGTGC
GGGGCGTGGCGCGGGGCTCGCCGTGCCGGGCGGGGGGTGGCGGCAGGTGGGGGTGCCGGGCGGGGCGGGGCCGCCTCGGGCCGGGGAGGGCTCGG
GGGAGGGGCGCGGCGGCCCCGGAGCGCCGGCGGCTGTCGAGGCGCGGCGAGCCGCAGCCATTGCCTTTTATGGTAATCGTGCGAGAGGGCGCAGGGA
CTTCCTTTGTCCCAAATCTGGCGGAGCCGAAATCTGGGAGGCGCCGCCGCACCCCCTCTAGCGGGCGCGGGCGAAGCGGTGCGGCGCCGGCAGGAAGG
AAATGGGCGGGGAGGGCCTTCGTGCGTCGCCGCGCCGCCGTCCCCTTCTCCATCTCCAGCCTCGGGGCTGCCGCAGGGGGACGGCTGCCTTCGGGGGGG
ACGGGGCAGGGCGGGGTTCGGCTTCTGGCGTGTGACCGGCGGCTCTAGAGCCTCTGCTAACCATGTTCATGCCTTCTTCTTTTTCCTACAGATCCTTAATTA
ATAATACGACTCACTATAGGGGGTCGACCCGCCACCAATCATTTAAATCACCATTCACGACACCGAAAAGTACGCCTCTCAGCCAGAACTATGCACTAACT
CCACTTGCATCGGATCTCGAGGACCTGGCTTTAGAGCCTTGGAGCACACCAAATACTCCTGTTGCGGGCACTGCAGAAACCCAGAACACTGGGGAAGCTG
GTTCCAAAGCCTGCCAAGATGGTCAACTGAGCCCAACTTGGTCAGAGATCGAGGAGGATTTGAGAGCGTGCTTCGGTGCGGAACCGTTGAAGAAAGACT
TCAGCGAGCCGCTGAACTTGGACTAAGGTACGATGGCGCCTCCAGCTAAAAGAGCTAAAAGAGGTAAGGGTTTAAGGGATGGTTGGTTGGTGGGGTATT
AATGTTTAATTACCTGTTTTACAGGCCTGAAATCACTTGGTTTTAGGTTGGGTGCCTCCTGGCTACAAGTACCTGGGACCAGGGAACAGCCTTGACCAAGG
AGAACCAACCAATCCATCTGACGCCGCTGCCAAAGAGCACGACGAGGCCTATGATCAATACATCAAATCTGGAAAAAATCCTTACCTGTACTTCTCTGCTG
CTGATCAACGCTTTATTGACCAAACCAAGGACGCCAAAGACTGGGGAGGCAAGGTTGGTCACTACTTTTTTAGAACCAAGCGCGCTTTTGCACCTAAGCTT
GCTACTGACTCTGAACCTGGAACTTCTGGTGTAAGCAGAGCTGGTAAACGCACTAGACCACCTGCTTACATTTTTATTAACCAAGCCAGAGCTAAAAAAAA
ACTTACTTCTTCTGCTGCACAGCAAAGCAGTCAAACCATGAGTGATGGCACCAGCCAACCTGACAGCGGAAACGCTGTCCACTCAGCTGCAAGAGTTGAA
CGAGCAGCTGACGGCCCTGGAGGCTCTGGGGGTGGGGGCTCTGGCGGGGGTGGGGTTGGTGTTTCTACTGGGTCTTATGATAATCAAACGCATTATAGA
TTCTTGGGTGACGGCTGGGTAGAAATTACTGCACTAGCAACTAGACTAGTACATTTAAACATGCCTAAATCAGAAAACTATTGCAGAATCAGAGTTCACAA
TACAACAGACACATCAGTCAAAGGCAACATGGCAAAAGATGATGCTCATGAGCAAATTTGGACACCATGGAGCTTGGTGGATGCTAATGCTTGGGGAGT
TTGGCTCCAGCCAAGTGACTGGCAATACATTTGCAACACCATGAGCCAGCTTAACTTGGTATCACTTGATCAAGAAATATTCAATGTAGTGCTGAAAACTG
TTACAGAGCAAGACTTAGGAGGTCAAGCTATAAAAATATACAACAATGACCTTACAGCTTGCATGATGGTTGCAGTAGACTCAAACAACATTTTGCCATAC
ACACCTGCAGCAAACTCAATGGAAACACTTGGTTTCTACCCCTGGAAACCAACCATAGCATCACCATACAGGTACTATTTTTGCGTTGACAGAGATCTTTCA
GTGACCTACGAAAATCAAGAAGGCACAGTTGAACATAATGTGATGGGAACACCAAAAGGAATGAATTCTCAATTTTTTACCATTGAGAACACACAACAAA
TCACATTGCTCAGAACAGGGGACGAATTTGCCACAGGTACTTACTACTTTGACACAAATTCAGTTAAACTCACACACACGTGGCAAACCAACCGTCAACTT
GGACAGCCTCCACTGCTGTCAACCTTTCCTGAAGCTGACACTGATGCAGGTACACTTACTGCTCAAGGGAGCAGACATGGAACAACACAAATGGGGGTTA
ACTGGGTGAGTGAAGCAATCAGAACCAGACCTGCTCAAGTAGGATTTTGTCAACCACACAATGACTTTGAAGCCAGCAGAGCTGGACCATTTGCTGCCCC
AAAAGTTCCAGCAGATATTACTCAAGGAGTAGACAAAGAAGCCAATGGCAGTGTTAGATACAGTTATGGCAAACAGCATGGTGAAAATTGGGCTTCACAT
GGACCAGCACCAGAGCGCTACACATGGGATGAAACAAGCTTTGGTTCAGGTAGAGACACCAAAGATGGTTTTATTCAATCAGCACCACTAGTTGTTCCAC
CACCACTAAATGGCATTCTTACAAATGCAAACCCTATTGGGACTAAAAATGACATTCATTTTTCAAATGTTTTTAACAGCTATGGTCCACTAACTGCATTTTC
ACACCCAAGTCCTGTATACCCTCAAGGACAAATATGGGACAAAGAACTAGATCTTGAACACAAACCTAGACTTCACATAACTGCTCCATTTGTTTGTAAAA
ACAATGCACCTGGACAAATGTTGGTTAGATTAGGACCAAACCTAACTGACCAATATGATCCAAACGGAGCCACACTTTCTAGAATTGTTACATACGGTACA
TTTTTCTGGAAAGGAAAACTAACCATGAGAGCAAAACTTAGAGCTAACACCACTTGGAACCCAGTGTACCAAGTAAGTGCTGAAGACAATGGCAACTCAT
ACATGAGTGTAACTAAATGGTTACCAACTGCTACTGGAAACATGCAGTCTGTGCCGCTTATAACAAGACCTGTTGCTAGAAATACTTACTAAGAGCTCGCT
GATCAGCCTCGACTGTGCCTTCTAGTTGCCAGCCATCTGTTGTTTGCCCCTCCCCCGTGCCTTCCTTGACCCTGGAAGGTGCCACTCCCACTGTCCTTTCCTA
ATAAAATGAGGAAATTGCATCGCATTGTCTGAGTAGGTGTCATTCTATTCTGGGGGGTGGGGTGGGGCAGGACAGCAAGGGGGAGGATTGGGAAGACA
ATAGCAGGCATGCTGGGGATGCGGTGGGCTCTATGGCTTCTGAGGCGGAAAGAACCAGCTGGGGCTCGAGATCCACTAGTTCTAGCCTCGAGGCTAGAG
CGGCCGCCACTGGCCGTCGTTTTACAACGTCGTGACTGGGAAAACCCTGGCGTTACCCAACTTAATCGCCTTGCAGCACATCCCCCTTTCGCCAGCTGGCG

TAATAGCGAAGAGGCCCGCACCGATCGCCCTTCCCAACAGTTGCGCAGCCTGAATGGCGAATGGGACGCGCCCTGTAGCGGCGCATTAAGCGCGGCGGG
TGTGGTGGTTACGCGCAGCGTGACCGCTACACTTGCCAGCGCCCTAGCGCCCGCTCCTTTCGCTTTCTTCCCTTCCTTTCTCGCCACGTTCGCCGGCTTTCCC
CGTCAAGCTCTAAATCGGGGGCTCCCTTTAGGGTTCCGATTTAGTGCTTTACGGCACCTCGACCCCAAAAAACTTGATTAGGGTGATGGTTCACGTAGTGG
GCCATCGCCCTGATAGACGGTTTTTCGCCCTTTGACGTTGGAGTCCACGTTCTTTAATAGTGGACTCTTGTTCCAAACTGGAACAACACTCAACCCTATCTC
GGTCTATTCTTTTGATTTATAAGGGATTTTGCCGATTTCGGCCTATTGGTTAAAAAATGAGCTGATTTAACAAAAATTTAACGCGAATTTTAACAAAATATTA
ACGCTTACAATTTAGGTGGCACTTTTCGGGGAAATGTGCGCGGAACCCCTATTTGTTTATTTTTCTAAATACATTCAAATATGTATCCGCTCATGAGACAAT
AACCCTGATAAATGCTTCAATAATATTGAAAAAGGAAGAGTATGAGTATTCAACATTTCCGTGTCGCCCTTATTCCCTTTTTTGCGGCATTTTGCCTTCCTGT
TTTTGCTCACCCAGAAACGCTGGTGAAAGTAAAAGATGCTGAAGATCAGTTGGGTGCACGAGTGGGTTACATCGAACTGGATCTCAACAGCGGTAAGATC
CTTGAGAGTTTTCGCCCCGAAGAACGTTTTCCAATGATGAGCACTTTTAAAGTTCTGCTATGTGGCGCGGTATTATCCCGTATTGACGCCGGGCAAGAGCA
ACTCGGTCGCCGCATACACTATTCTCAGAATGACTTGGTTGAGTACTCACCAGTCACAGAAAAGCATCTTACGGATGGCATGACAGTAAGAGAATTATGCA
GTGCTGCCATAACCATGAGTGATAACACTGCGGCCAACTTACTTCTGACAACGATCGGAGGACCGAAGGAGCTAACCGCTTTTTTGCACAACATGGGGGA
TCATGTAACTCGCCTTGATCGTTGGGAACCGGAGCTGAATGAAGCCATACCAAACGACGAGCGTGACACCACGATGCCTGTAGCAATGGCAACAACGTTG
CGCAAACTATTAACTGGCGAACTACTTACTCTAGCTTCCCGGCAACAATTAATAGACTGGATGGAGGCGGATAAAGTTGCAGGACCACTTCTGCGCTCGG
CCCTTCCGGCTGGCTGGTTTATTGCTGATAAATCTGGAGCCGGTGAGCGTGGGTCTCGCGGTATCATTGCAGCACTGGGGCCAGATGGTAAGCCCTCCCG
TATCGTAGTTATCTACACGACGGGGAGTCAGGCAACTATGGATGAACGAAATAGACAGATCGCTGAGATAGGTGCCTCACTGATTAAGCATTGGTAACTG
TCAGACCAAGTTTACTCATATATACTTTAGATTGATTTAAAACTTCATTTTTAATTTAAAAGGATCTAGGTGAAGATCCTTTTTGATAATCTCATGACCAAAA
TCCCTTAACGTGAGTTTTCGTTCCACTGAGCGTCAGACCCCGTAGAAAAGATCAAAGGATCTTCTTGAGATCCTTTTTTTCTGCGCGTAATCTGCTGCTTGCA
AACAAAAAAACCACCGCTACCAGCGGTGGTTTGTTTGCCGGATCAAGAGCTACCAACTCTTTTTCCGAAGGTAACTGGCTTCAGCAGAGCGCAGATACCA
AATACTGTCCTTCTAGTGTAGCCGTAGTTAGGCCACCACTTCAAGAACTCTGTAGCACCGCCTACATACCTCGCTCTGCTAATCCTGTTACCAGTGGCTGCT
GCCAGTGGCGATAAGTCGTGTCTTACCGGGTTGGACTCAAGACGATAGTTACCGGATAAGGCGCAGCGGTCGGGCTGAACGGGGGGTTCGTGCACACA
GCCCAGCTTGGAGCGAACGACCTACACCGAACTGAGATACCTACAGCGTGAGCTATGAGAAAGCGCCACGCTTCCCGAAGGGAGAAAGGCGGACAGGT
ATCCGGTAAGCGGCAGGGTCGGAACAGGAGAGCGCACGAGGGAGCTTCCAGGGGGAAACGCCTGGTATCTTTATAGTCCTGTCGGGTTTCGCCACCTCT
GACTTGAGCGTCGATTTTTGTGATGCTCGTCAGGGGGGCGGAGCCTATGGAAAAACGCCAGCAACGCGGCCTTTTTACGGTTCCTGGCCTTTTGCTGGCC
TTTTGCTCACATGTTCTTTCCTGCGTTATCCCCTGATTCTGTGGATAACCGTATTACCGCCTTTGAGTGAGCTGATACCGCTCGCCGCAGCCGAACGACCGA
GCGCAGCGAGTCAGTGAGCGAGGAAGCGGAAGAGCGCCCAATACGCAAACCGCCTCTCCCCGCGCGTTGGCCGATTCATTAATGCAGCTGGCACGACAG
GTTTCCCGACTGGAAAGCGGGCAGTGAGCGCAACGCAATTAATGTGAGTTAGCTCACTCATTAGGCACCCCAGGCTTTACACTTTATGCTTCCGGCTCGTA
TGTTGTGTGGAATTGTGAGCGGATAACAATTTCACACAGGAAACAGCTATGACCATGAGGCGCGCCG

C. In vitro translation plasmid

MVM region underlined, insert region highlighted in grey

>pMKRQ_MVMp_synth

CTAAATTGTAAGCGTTAATATTTTGTTAAAATTCGCGTTAAATTTTTGTTAAATCAGCTCATTTTTTAACCAATAGGCCGAAATCGGCAAAATCCCTTATAAA
TCAAAAGAATAGACCGAGATAGGGTTGAGTGGCCGCTACAGGGCGCTCCCATTCGCCATTCAGGCTGCGCAACTGTTGGGAAGGGCGTTTCGGTGCGGG
CCTCTTCGCTATTACGCCAGCTGGCGAAAGGGGGATGTGCTGCAAGGCGATTAAGTTGGGTAACGCCAGGGTTTTCCCAGTCACGACGTTGTAAAACGAC
GGCCAGTGAGCGCGACGTAATACGACTCACTATAGGGCGAATTGAAGGAAGGCCGTCAAGGCCGCATCCTAAGCTTGCTACTGACTCTGAACCTGGAACT
TCTGGTGTAAGCAGAGCTGGTAAACGCACTAGACCACCTGCTTACATTTTTATTAACCAAGCCAGAGCTAAAAAAAAACTTACTTCTTCTGCTGCACAGCAA
AGCAGTCAAACCATGAGTGATGGCACCAGCCAACCTGACAGCGGAAACGCTGTCCACTCAGCTGCAAGAGTTGAACGAGCAGCTGACGGCCCTGGAGGC
TCTGGGGGTGGGGGCTCTGGCGGGGGTGGGGTTGGTGTTTCTACTGGGTCTTATGATAATCAAACGCATTATAGATTCTTGGGTGACGGCTGGGTAGAA
ATTACTGCACTAGCAACTAGACTAGTACATTTAAACATGCCTAAATCAGAAAACTATTGCAGAATCAGAGTTCACAATACAACAGACACATCAGTCAAAGG
CAACATGGCCAAAGATGATGCACATGAGCAAATTTGGACACCATGGAGCTTGGTTGATGCAAATGCGTGGGGAGTTTGGCTTCAACCAAGTGACTGGCA
ATACATTTGCAACACAATGAGTCAACTTAATTTGGTTTCACTTGATCAAGAAATATTCAATGTAGTGTTGAAAACTGTGACAGAGCAAGACTTAGGAGGAC
AAGCTATAAAAATATACAACAATGACCTCACAGCATGTATGATGGTAGCAGTAGATTCTAACAACATATTGCCATACACTCCAGCAGCAAACTCAATGGAA
ACTCTTGGATTCTACCCATGGAAACCTACTATTGCTTCCCCATACAGATACTATTTTTGCGTTGACAGAGACCTATCAGTGACATATGAAAATCAAGAAGGC
ACTGTTGAACATAATGTCATGGGAACACCAAAAGGAATGAACTCACAATTTTTTACCATTGAGAACACACAGCAAATCACCTTGCTGAGAACTGGGGACG
AATTTGCCACAGGCACCTACTACTTTGACACAAATTCAGTTAAACTTACACACACTTGGCAAACAAACCGTCAACTAGGACAGCCTCCACTTCTTTCAACATT
TCCTGAAGCAGACACTGATGCAGGTACACTAACTGCACAAGGAAGCAGACATGGGACAACTCAAATGGGAGTTAACTGGGTTAGTGAAGCCATAAGAAC

CAGACCAGCTCAAGTAGGCTTTTGTCAGCCACACAATGACTTTGAAGCAAGCAGGGCAGGCCCATTTGCAGCACCTAAAGTTCCAGCTGACATTACACAA
GGAGTGGACAAAGAAGCCAATGGTAGTGTTAGATACAGCTATGGCAAGCAGCATGGTGAAAATTGGGCTTCACATGGACCTGCACCTGAACGTTACACA
TGGGATGAAACAAGTTTTGGTTCTGGCAGAGACACAAAAGACGGCTTCATTCAATCTGCACCACTAGTTGTGCCACCACCTCTAAATGGCATCCTTACCAA
TGCAAATCCAATTGGGACAAAAAATGACATTCACTTTTCAAATGTTTTTAACAGTTATGGACCTCTAACTGCTTTTTCACACCCAAGTCCAGTGTACCCTCAA
GGTCAAATATGGGATAAAGAACTAGATCTTGAACACAAACCCAGACTTCACATTACTGCACCCTTTGTTTGTAAAAACAATGCACCTGGACAAATGTTGGT
TAGGCTAGGACCCAACCTTACAGACCAATATGATCCCAACGGAGCAACACTTTCTAGACTGGGCCTCATGGGCCTTCCTTTCACTGCCCGCTTTCCAGTCGG
GAAACCTGTCGTGCCAGCTGCATTAACATGGTCATAGCTGTTTCCTTGCGTATTGGGCGCTCTCCGCTTCCTCGCTCACTGACTCGCTGCGCTCGGTCGTTC
GGGTAAAGCCTGGGGTGCCTAATGAGCAAAAGGCCAGCAAAAGGCCAGGAACCGTAAAAAGGCCGCGTTGCTGGCGTTTTTCCATAGGCTCCGCCCCCC
TGACGAGCATCACAAAAATCGACGCTCAAGTCAGAGGTGGCGAAACCCGACAGGACTATAAAGATACCAGGCGTTTCCCCCTGGAAGCTCCCTCGTGCGC
TCTCCTGTTCCGACCCTGCCGCTTACCGGATACCTGTCCGCCTTTCTCCCTTCGGGAAGCGTGGCGCTTTCTCATAGCTCACGCTGTAGGTATCTCAGTTCGG
TGTAGGTCGTTCGCTCCAAGCTGGGCTGTGTGCACGAACCCCCCGTTCAGCCCGACCGCTGCGCCTTATCCGGTAACTATCGTCTTGAGTCCAACCCGGTA
AGACACGACTTATCGCCACTGGCAGCAGCCACTGGTAACAGGATTAGCAGAGCGAGGTATGTAGGCGGTGCTACAGAGTTCTTGAAGTGGTGGCCTAAC
TACGGCTACACTAGAAGAACAGTATTTGGTATCTGCGCTCTGCTGAAGCCAGTTACCTTCGGAAAAAGAGTTGGTAGCTCTTGATCCGGCAAACAAACCAC
CGCTGGTAGCGGTGGTTTTTTTGTTTGCAAGCAGCAGATTACGCGCAGAAAAAAAGGATCTCAAGAAGATCCTTTGATCTTTTCTACGGGGTCTGACGCTC
AGTGGAACGAAAACTCACGTTAAGGGATTTTGGTCATGAGATTATCAAAAAGGATCTTCACCTAGATCCTTTTAAATTAAAAATGAAGTTTTAAATCAATCT
AAAGTATATATGAGTAAACTTGGTCTGACAGTTATTAGAAAAATTCATCCAGCAGACGATAAAACGCAATACGCTGGCTATCCGGTGCCGCAATGCCATAC
AGCACCAGAAAACGATCCGCCCATTCGCCGCCCAGTTCTTCCGCAATATCACGGGTGGCCAGCGCAATATCCTGATAACGATCCGCCACGCCCAGACGGC
CGCAATCAATAAAGCCGCTAAAACGGCCATTTTCCACCATAATGTTCGGCAGGCACGCATCACCATGGGTCACCACCAGATCTTCGCCATCCGGCATGCTC
GCTTTCAGACGCGCAAACAGCTCTGCCGGTGCCAGGCCCTGATGTTCTTCATCCAGATCATCCTGATCCACCAGGCCCGCTTCCATACGGGTACGCGCACG
TTCAATACGATGTTTCGCCTGATGATCAAACGGACAGGTCGCCGGGTCCAGGGTATGCAGACGACGCATGGCATCCGCCATAATGCTCACTTTTTCTGCCG
GCGCCAGATGGCTAGACAGCAGATCCTGACCCGGCACTTCGCCCAGCAGCAGCCAATCACGGCCCGCTTCGGTCACCACATCCAGCACCGCCGCACACGG
AACACCGGTGGTGGCCAGCCAGCTCAGACGCGCCGCTTCATCCTGCAGCTCGTTCAGCGCACCGCTCAGATCGGTTTTCACAAACAGCACCGGACGACCC
TGCGCGCTCAGACGAAACACCGCCGCATCAGAGCAGCCAATGGTCTGCTGCGCCCAATCATAGCCAAACAGACGTTCCACCCACGCTGCCGGGCTACCCG
CATGCAGGCCATCCTGTTCAATCATACTCTTCCTTTTTCAATATTATTGAAGCATTTATCAGGGTTATTGTCTCATGAGCGGATACATATTTGAATGTATTTA
GAAAAATAAACAAATAGGGGTTCCGCGCACATTTCCCCGAAAAGTGCCAC

TABLE S2

Accession numbers of autonomous parvoviruses analysed for CpG and UpA composition*

AY386330, J02275, M19296, M14363, M20036, L23427, AJ249437, AF495467, AF406966, AY622943, DQ000496, FJ170278, EU918736, FJ973561, HM145750, GQ387499, JF429834, JF429835, JN831651, JQ037753, JQ037754, JQ692585, JN648103, KC617868, KC992732, KF792837, JX896321, KF360033, KF999685, GU214704, HM053693, GU214705, AB847987, KJ396347, KM926355, AB937988, KT225725, KT716186, KP729195, KT454512, KT592508, LC085675, KU172421, KU950356, KT878837, KU321655, KT878839, KX981923, KX517759, KY640438, KY640421, MG026727, JN202450, GU214706, JN420361, JN420365, HQ223038, HQ291308, U26342, AF221122, AF221123, GQ200736, JX027296, AF036710, EU200677, JF504699, GU938300, DQ335247, EU200669, KM017744, JQ814850, KC339251, KC339250, KU321654, JX627576 and KT868811.

*Parvovirus sequences were obtained from the Refseq database on the 23rd September, 2018. Sequences of the coding regions of 34,197 human mRNA sequences longer than 400 nucleotides in length were obtained from Refseq and composition calculated similarly.

FIGURE S1

Replication kinetics of MVM WT and compositionally altered mutants in 324K cells

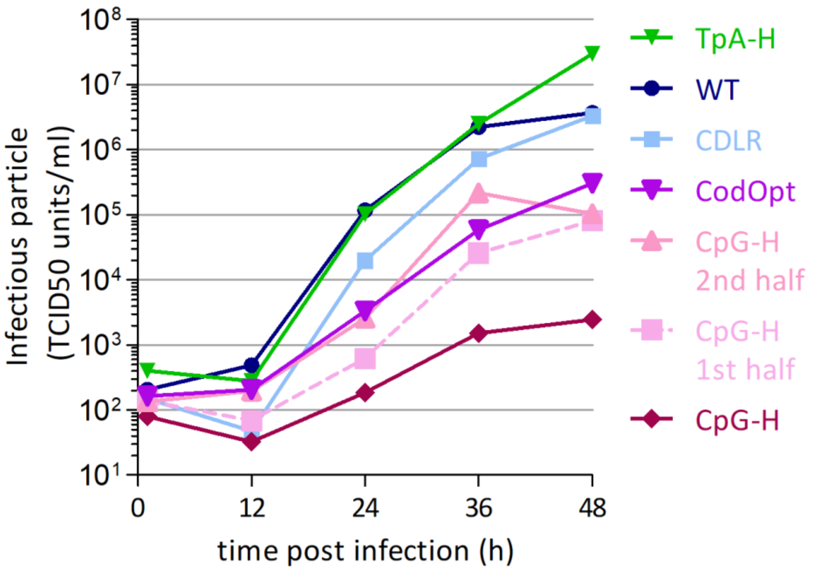

265 Growth curve in NB324K cell line, infected with 1 TCID50 unit/cell, titred in NB324K cells. Data points represent the mean of three biological replicates.

FIGURE S2

Cellular distribution of MVM viral proteins in A9 cells

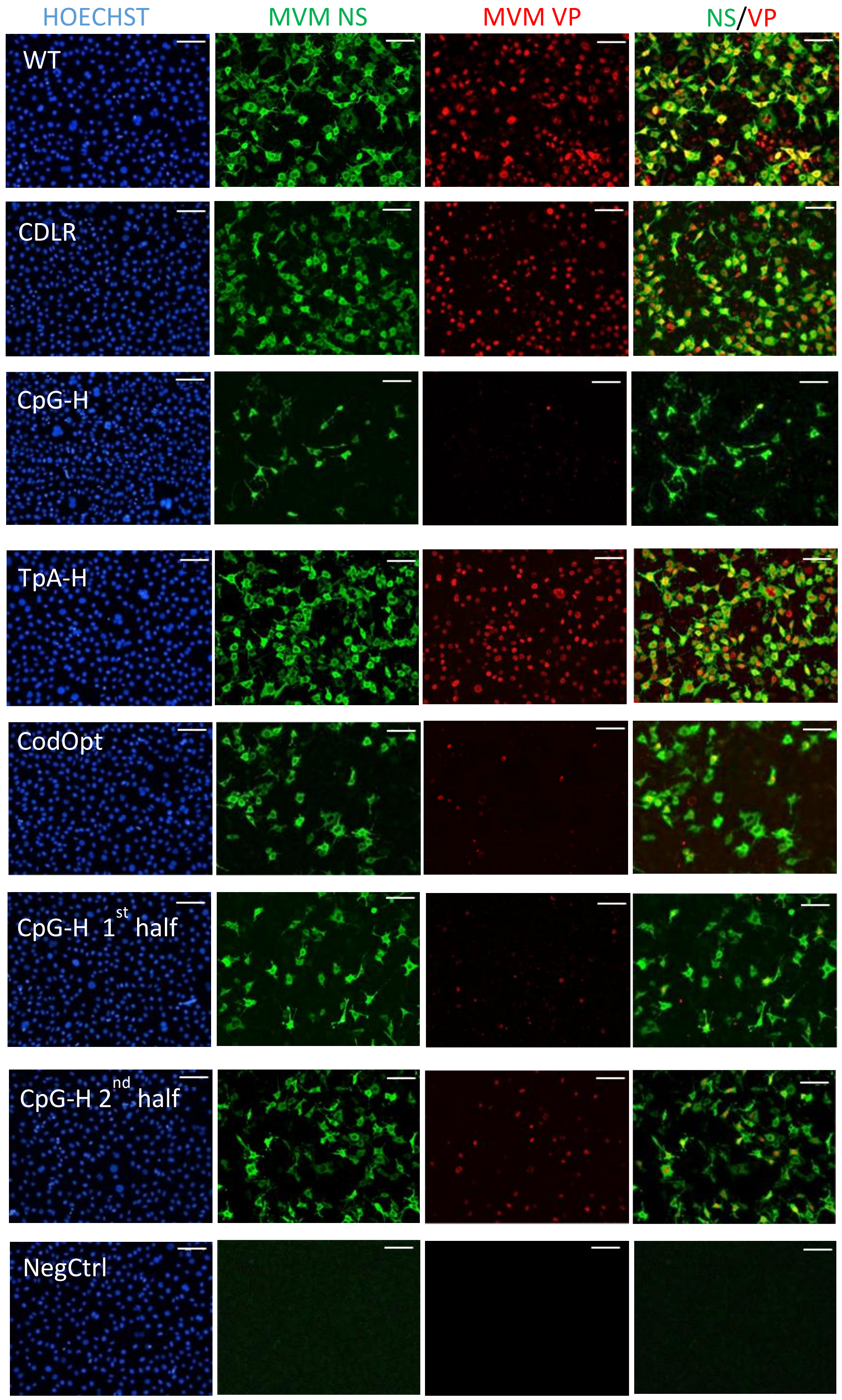

Immunofluorescence staining of viral NS (green) and VP (red) proteins. At 18 h post infected with 1000 viral copies/cell. Uninfected cells were used as a negative control, Hoechst 33342 was used as a nuclear stain. n = 3, scale bar 100 µm, representative image shown.

FIGURE S3

DNA / INFECTIVITY RATIOS OF WT AND CpG-MODIFIED MUTANTS OF MVM

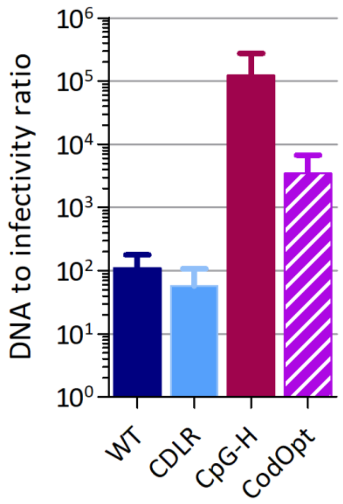

DNA to infectivity ratio of MVM WT and mutants. Virus titres were determined in infectivity titrations on NB324K cells; copy numbers were measured using qPCR. Bar heights represent the mean of three biological replicates; error bar show SEMs.

FIGURE S4

MVM sequence changes after serial passage

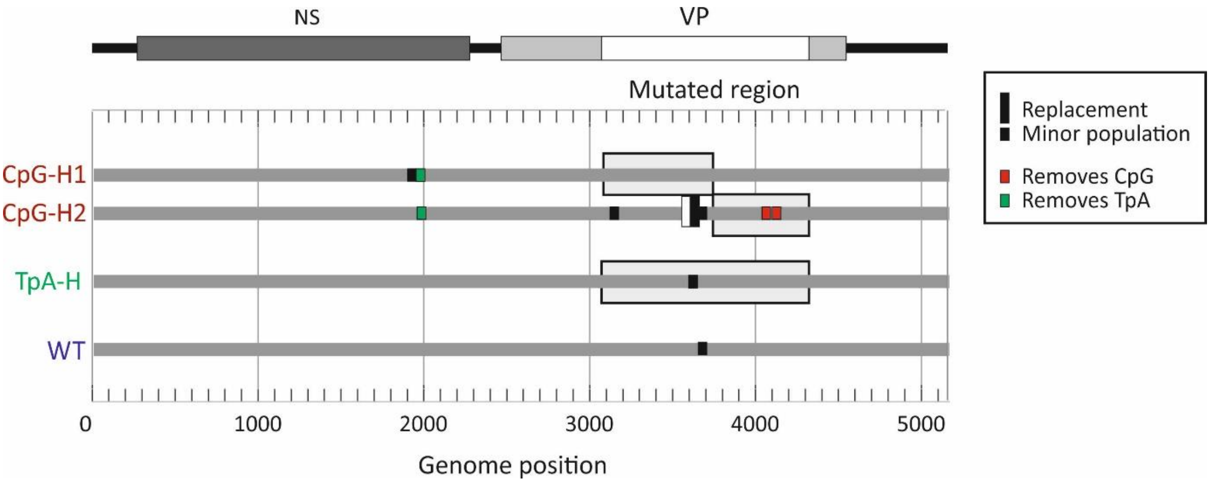

Positions of sequence changes in the MVM genome after serial passage of CpG-H mutants of MVM and non-attenuated WT and UpA-H controls in A9 cells. Tall symbols indicate complete replacement; short symbols indicate polymorphic populations with >10 % representation of mutated sequences.

FIGURE S5

Titres of MVM WT and compositionally altered mutants in different cell lines

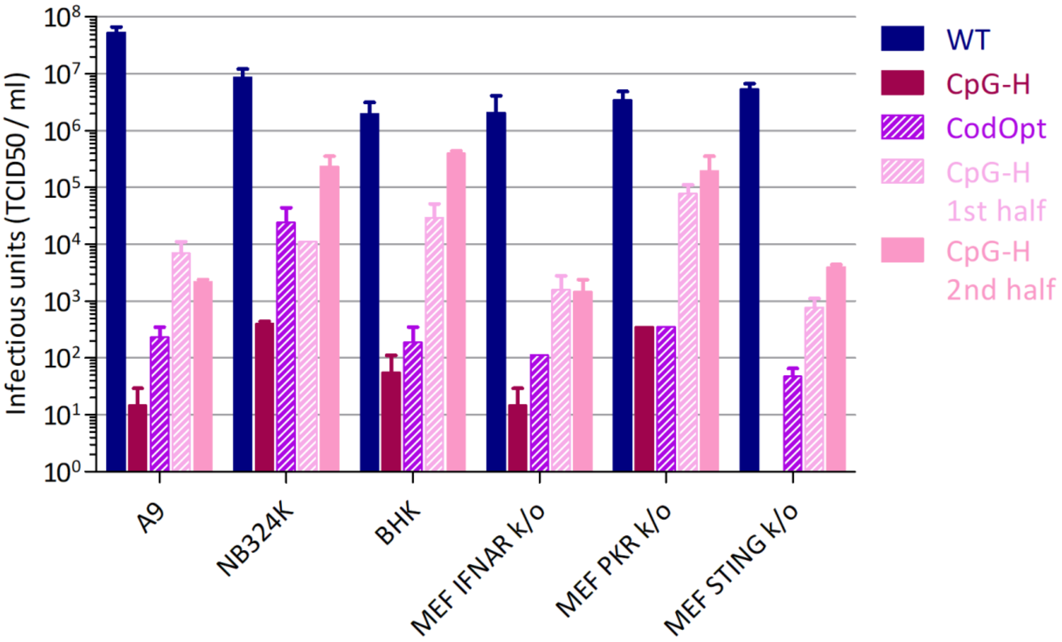

Infectious particles of MVM recovered from cell culture supernatant 72 h post infection (1 TCID unit / cell) in different cell lines. n = 2, +- SD.

FIGURE S6

Cellular distribution of MVM viral proteins in A549 and B8 (ZAP k/o) cells

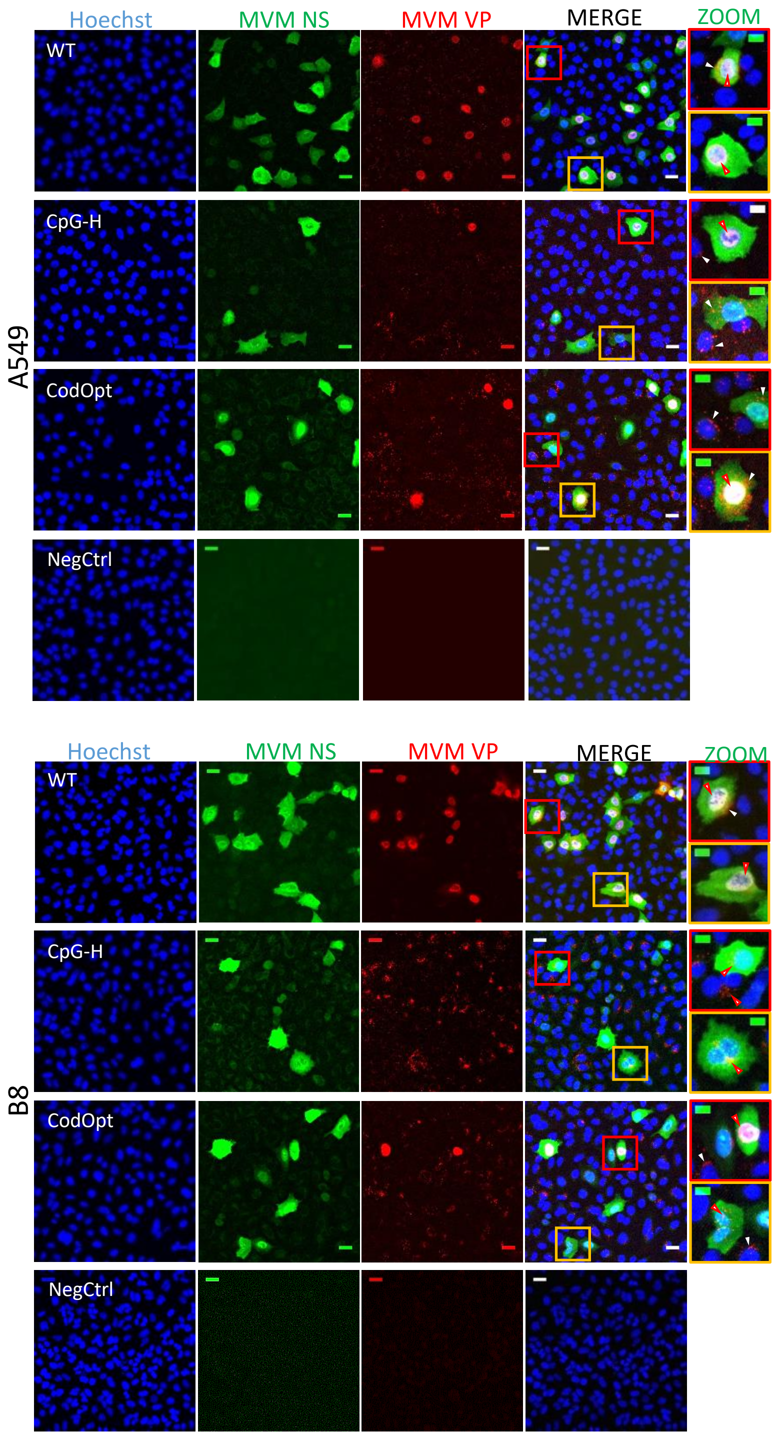

Immunofluorescence staining of viral NS (green) and VP (red) proteins. At 18 h post infected with 1000 viral copies/cell. Uninfected cells were used as a negative control, Hoechst 33342was used as a nuclear stain. n = 3, scale bar 100 µm, representative image shown.

FIGURE S7

Subcellular localisation of MVM viral proteins in A549 and B8 (ZAP k/o) cells

A549 WT

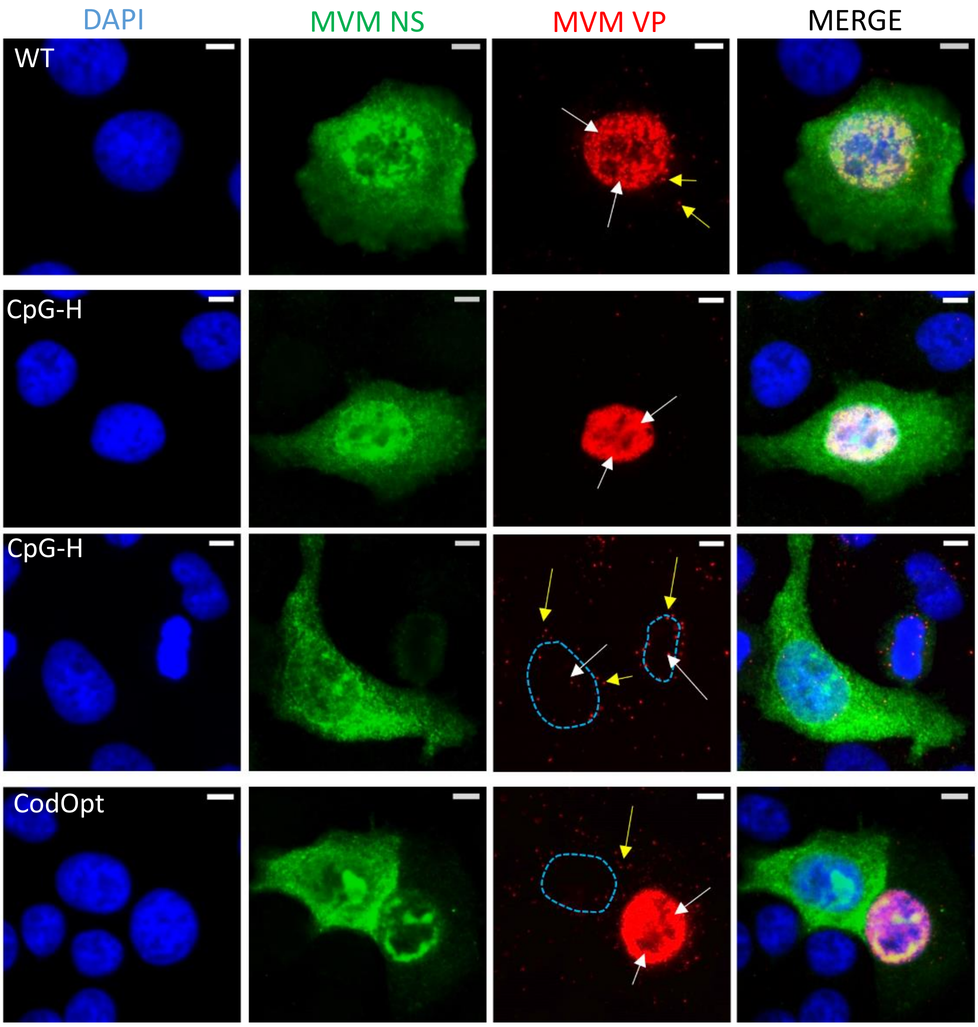

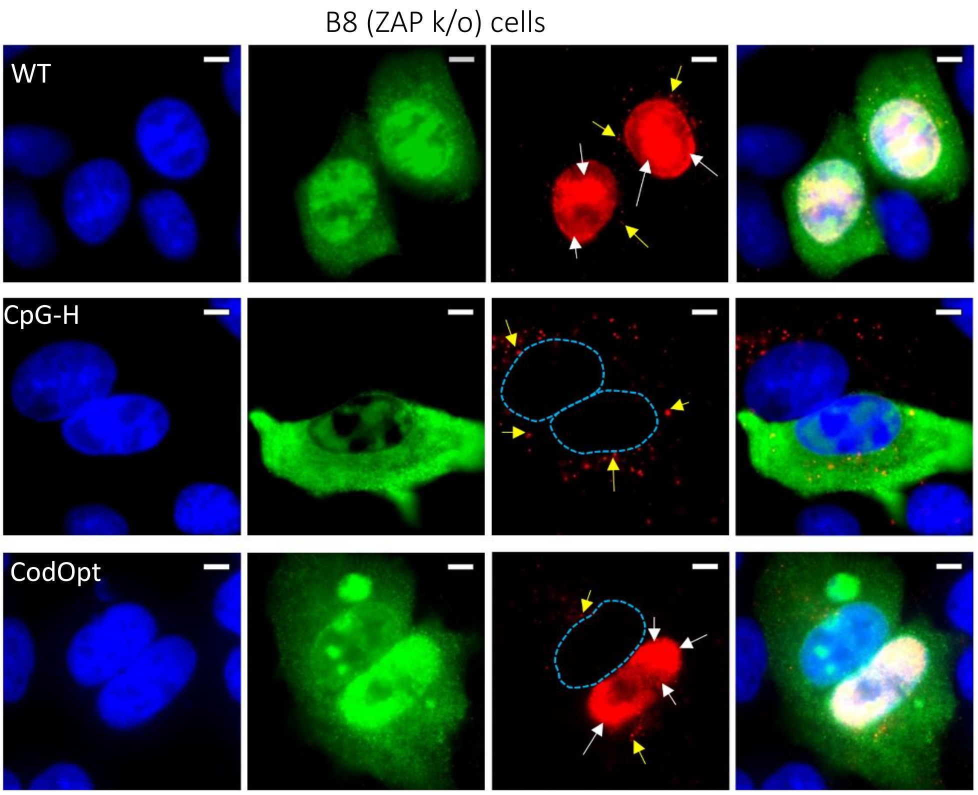

Immunofluorescence staining of viral NS (green) and VP (red) proteins. At 18 h post infected with 1000 viral copies/cell. Uninfected cells were used as a negative control, Hoechst 33342was used as a nuclear stain. n = 3, scale bar 10 µm, representative image shown.
